# Identification of N-linked glycans as specific mediators of neuronal uptake of acetylated α-Synuclein

**DOI:** 10.1371/journal.pbio.3000318

**Published:** 2019-06-18

**Authors:** Melissa Birol, Slawomir P. Wojcik, Andrew D. Miranker, Elizabeth Rhoades

**Affiliations:** 1 Department of Chemistry, University of Pennsylvania, Philadelphia, Pennsylvania, United States of America; 2 Department of Molecular Biophysics and Biochemistry, Yale University, New Haven, Connecticut, United States of America; 3 Biochemistry and Molecular Biophysics Graduate Group, University of Pennsylvania, Philadelphia, Pennsylvania, United States of America; University College London Institute of Neurology, UNITED KINGDOM

## Abstract

Cell-to-cell transmission of toxic forms of α-Synuclein (αS) is thought to underlie disease progression in Parkinson disease. αS in humans is constitutively N-terminally acetylated (αS_acetyl_), although the impact of this modification is relatively unexplored. Here, we report that αS_acetyl_ is more effective at inducing intracellular aggregation in primary neurons than unmodified αS (αS_un_). We identify complex N-linked glycans as binding partners for αS_acetyl_ and demonstrate that cellular internalization of αS_acetyl_ is reduced significantly upon cleavage of extracellular N-linked glycans, but not other carbohydrates. We verify binding of αS_acetyl_ to N-linked glycans in vitro, using both isolated glycans and cell-derived proteoliposomes. Finally, we identify neurexin 1β, a neuronal glycoprotein, as capable of driving glycan-dependent uptake of αS_acetyl_. Importantly, our results are specific to αS_acetyl_ because αS_un_ does not demonstrate sensitivity for N-linked glycans in any of our assays. Our study identifies extracellular N-linked glycans—and the glycoprotein neurexin 1β specifically—as key modulators of neuronal uptake of αS_acetyl_, drawing attention to the potential therapeutic value of αS_acetyl_-glycan interactions.

## Abstract 2

This study identifies extracellular N-linked glycans — and the glycoprotein neurexin 1β — as key modulators of neuronal uptake of N-terminally acetylated α-Synuclein, emphasising the potential therapeutic value of α-Synuclein-glycan interactions.

## Introduction

The pathologies of Parkinson disease and related synucleinopathies are characterized by the accumulation of aggregates of the neuronal protein α-Synuclein (αS) [1]. The prevailing hypothesis is that toxicity is mediated by prefibrillar oligomers of αS [2]. Emerging evidence suggests that cell-to-cell transmission of toxic αS species may be the basis of disease propagation [3].

αS is a small, soluble protein that is intrinsically disordered in the cytoplasm [4]. It associates peripherally to anionic lipid bilayers through its N-terminal domain, which becomes α-helical upon binding [5]. The localization of αS to nerve terminals [6,7] and to cellular lipid raft domains [8] suggests that there are components or properties of cellular membranes that are important for αS binding and function that may not be fully reproduced by simple lipid mixtures. Indeed, specific components of the extracellular membrane, including proteins [9] and proteoglycans [10], have been identified as having roles in the uptake of pathogenic αS species.

αS is subject to various post-translational modifications, including phosphorylation, ubiquitination, glycation, acetylation, and arginylation, some of which are correlated with pathology [11–13]. Mass spectrometry analysis indicates that the majority of these modifications are found on only a very small fraction of αS [14]. N-terminal acetylation, however, is unique in that it appears to be present on the majority of αS in vivo [11,15,16], in both healthy persons and patients with Parkinson disease [14,15]. Acetylation of the amino terminus occurs co-translationally [17], and, for many proteins, it is required for recognition of cellular binding partners [18]. Work from our group [19] and others have demonstrated that N-terminal acetylation alters the fundamental biophysical properties of αS; it moderately affects its binding to synthetic lipid bilayers [5,20] and rates of aggregation [20]. How this modification impacts interactions with other cellular binding partners—and in particular the plasma membrane proteins that have been identified as receptors involved in cellular uptake and aggregation—has not been explored. Here, we investigate the role of N-terminal acetylation of αS on cellular binding and internalization. Using cell biological and biophysical approaches, we demonstrate that N-terminal acetylation of αS confers interactions with extracellular N-linked glycans that impact cellular uptake, identify neurexin 1β as a receptor for glycan-dependent uptake of αS, and provide insight into the mechanism of cellular recognition relevant to uptake.

## Results

### N-terminal acetylation of αS enhances formation of intracellular aggregates in neurons

**Fig 1:**
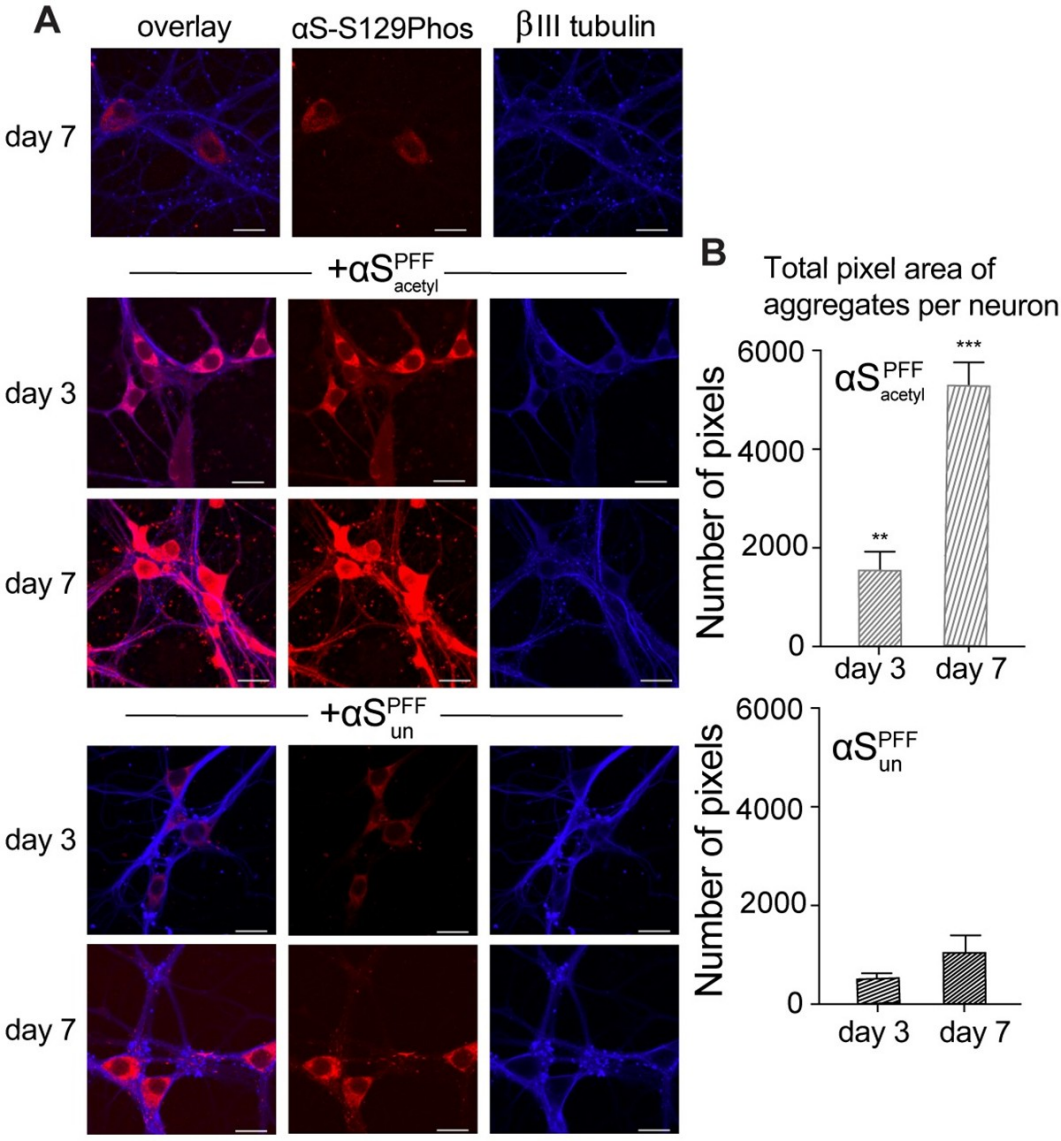
αS_acetyl_ PFFs are more effective at inducing pathological aggregates in primary neurons. (A) Representative images of aggregates of endogenous αS in cultured mouse hippocampal neurons following incubation with αS_acetyl_ or αS_un_ PFFs. Images shown at day 7 in the absence of PFFs (upper row) and after 3 and 7 days following treatment with PFFs. Red: αS-pS129 antibody; blue: βIII-tubulin antibody. (B) Quantification of αS aggregates as seen in images in (A). Aggregates in neurons treated with αS_acetyl_ PFFs are larger and more numerous. *n* = 80 neurons, 3 independent experiments, **P < 0.001 and ***P < 0.0001 by Student *t* test compared to αS_un_ PFF-treated neurons. The data are presented as mean ± SD, *n* = 3. Scale bars = 20 μm. The underlying data for this figure can be found in S1 Data. αS, α-Synuclein; αS-pS129, αS phosphorylated at serine 129; αS_acetyl_, N-terminally acetylated αS; αS_un_, unmodified αS; PFF, preformed fibril.

Recently, observations that exogenously added aggregates of αS are capable of seeding aggregation of endogenous αS have prompted an interest in understanding the molecular players involved. However, investigations of this phenomenon have relied on aggregates composed of unmodified αS (αS_un_). To determine whether N-terminal acetylation of αS (αS_acetyl_) alters this seeding behavior, primary hippocampal neurons were incubated with preformed fibrils (PFFs) of αS_acetyl_ or αS_un_ [21] (S1 Fig). Both αS_acetyl_ and αS_un_ PFFs resulted in the formation of abundant intracellular aggregates (Fig 1A), demonstrating that the internalized material retains its fibrillar character, as previously characterized [2]. However, the kinetics of aggregate formation differ significantly. For αS_un_ PFFs, the time course was in good agreement with previously published studies [21]; aggregates were observed in axons by day 7 and had spread to somatodendritic compartments by day 10. For αS_acetyl_ PFFs, aggregates in axons were already prevalent by day 3, and spreading to somatodentric compartments was readily apparent by day 7. Measurements beyond 7 days were not possible for neurons treated with αS_acetyl_ PFFs due to significant cell death.

To compare the effectiveness of αS_acetyl_ and αS_un_ PFFs in nucleating intracellular aggregate formation and growth, we quantified the total number of aggregates per neuron, reflecting the seeding capacity of the added αS PFFs (i.e., their ability to induce the initial formation of intracellular αS aggregates), as well as total aggregate area per neuron, reflecting the overall ability of the added αS PFFs to accelerate further aggregate growth. This quantification revealed that the overall rate of aggregate formation is >2-fold faster for neurons treated with αS_acetyl_ PFFs compared with αS_un_ PFFs (Fig 1B). This result demonstrates that αS_acetyl_ PFFs are markedly more potent seeds for pathological propagation of αS aggregation in neurons.

### αS_acetyl_ is more rapidly internalized by SH-SY5Y cells than αS_un_

**Fig 2:**
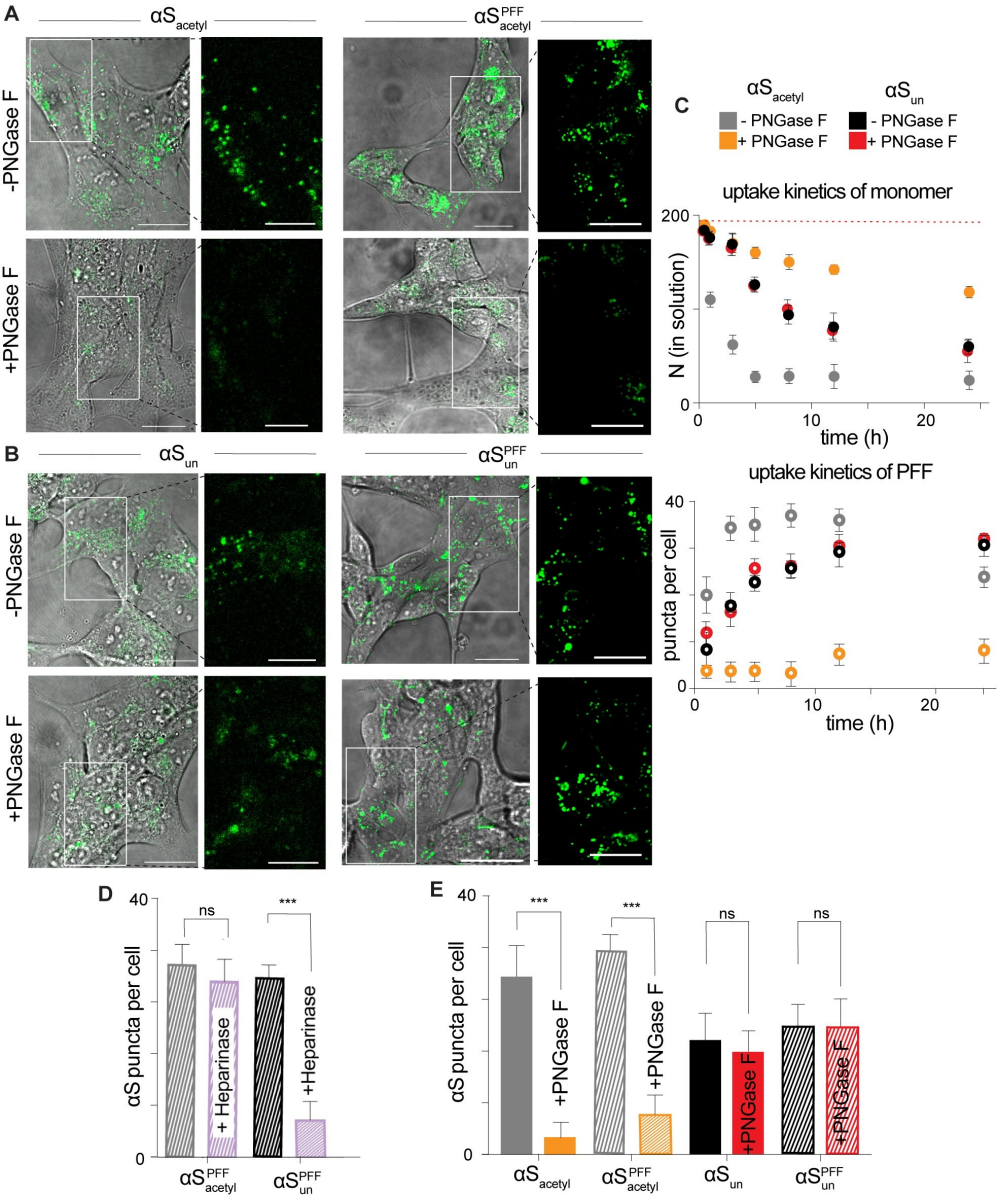
Complex N-linked glycans selectively enhance uptake of αS_acetyl_ by SH-SY5Y cells. (A) Representative images of SH-SY5Y cells following 12-hour incubation with monomer or PFF αS_acetyl_-AL488, −/+ PNGase F treatment. (B) As in (A) but for monomer or PFF αS_un_ -AL488. (C) Upper: kinetics of internalization by SH-SY5Y cells of monomer αS as quantified by loss from extracellular medium by FCS, −/+ PNGase F treatment. Lower: kinetics of internalization by SH-SY5Y cells of PFF αS as quantified by puncta analysis of confocal images, −/+ PNGase F treatment. (D) Quantification of internalization of αS PFFs by SH-SY5Y cells, −/+ Heparinase I/III treatment. Images collected following 12-hour incubation with protein and quantified by puncta analysis. (E) Quantification of internalization of αS monomer and PFF by SH-SY5Y cells, −/+ PNGase F treatment. Images collected following 12-hour incubation with protein and quantified by puncta analysis. All protein uptake measurements with 200 nM monomer or 200 nM PFFs (monomer units, 20:1 αS:αS-AL488); *n* = 100 cells, 3 independent experiments, ***P < 0.0001 by Student *t* test). Scale bars = 20 μm. The underlying data for this figure can be found in S1 Data. αS, α-Synuclein; αS_acetyl_, N-terminally acetylated αS; αS_un_, unmodified αS; AL488, Alexa Fluor 488; FCS, fluorescence correlation spectroscopy; PFF, preformed fibril; PNGase F, peptide-N-glycosidase F; SH-SY5Y, human neuroblastoma.

This observation sparked our interest in determining the origin in differences in aggregate propagation in neurons. To do so, human neuroblastoma (SH-SY5Y) cells were chosen because they retain many of the pathways dysregulated in Parkinson disease and thus are widely used as a cellular model for disease. Moreover, SH-SY5Y cells spontaneously internalize αS without requiring the use of a transfection agent [22]. SH-SY5Y cells were incubated with monomer or PFF αS fluorescently labeled with Alexa Fluor 488 (αS-AL488). After 12 hours of incubation, monomer and PFF αS_acetyl_ and αS_un_ appeared as puncta, colocalized with an endosomal marker (Fig 2A and 2B, S2A and S2B Fig). In order to make a more quantitative comparison of both kinetics and quantity of uptake between αS_acetyl_ and αS_un_, cellular internalization was measured as a function of time. Three orthogonal methods were used to quantify uptake: (1) loss of monomer αS from the cell media was measured using fluorescence correlation spectroscopy (FCS) [23], (2) the amounts of internalized monomer and PFF αS were quantified by confocal imaging, or (3) both extracellular and internalized monomer and PFF αS were quantified by polyacrylamide gel electrophoresis (S3 Fig). Results of all three of these approaches are consistent and reveal that both monomer and PFF αS_acetyl_ are internalized more rapidly (Fig 2C) and to a greater extent (Fig 2D, S3 Fig) than αS_un_. Uptake was inhibited at 4°C, indicating that active endocytotic pathways are required (S4A Fig). Control measurements made using non-neuronal lineage human embryonic kidney 293T (HEK) cells found no spontaneous internalization of either αS_acetyl_ or αS_un_ (S4B Fig), consistent with prior studies of αS uptake by HEK cells that utilized transfection agents [24–26]. Both SH-SY5Y and HEK cells showed rapid uptake of transferrin, indicating that lack of αS internalization is not due to inherent differences in rates of clathrin-dependent endocytosis between the cell lines (S4C Fig).

### Cleavage of extracellular N-linked glycans inhibits uptake of αS_acetyl_ by SH-SY5Y cells

Cell-surface heparan sulfate proteoglycans have been observed to promote uptake of a number of fibrillar amyloid proteins, including αS_un_ PFFs [27]. To investigate the relevance of proteoglycans to αS_acetyl_ uptake, SH-SY5Y cells were treated with Heparinase I/III, an enzyme that cleaves these carbohydrates, for 6 hours. After exchange of media to remove the enzyme, αS-AL488 was added and incubated an additional 12 hours. This incubation period was chosen because it allows for reproducible quantification of puncta, and there is no evidence of protein or fluorophore degradation that may occur at longer time points (S2B Fig). In agreement with previous reports, we found that treatment of SH-SY5Y cells with Heparinase reduced the uptake of αS_un_ PFFs (Fig 2D, S5A Fig). Interestingly, however, Heparinase pretreatment did not alter uptake of αS_acetyl_ PFFs nor that of αS_acetyl_ monomer (Fig 2D, S5A and S5B Fig).

These results prompted us to consider other endoglycosidases, as the majority of cell-surface proteins are modified by glycosylation [28], including a number of proteins that have been identified as receptors for αS_un_ PFFs [9,29,30]. Complex N-linked glycans were selectively removed from SH-SY5Y cells using peptide-N-glycosidase F (PNGase F). Following incubation with αS_acetyl_, a decrease in the number of intracellular puncta in the PNGase F–treated cells relative to untreated cells was observed, both for monomer and PFF αS_acetyl_, reflecting a significant decrease in uptake by deglycosylated cells (Fig 2A, 2C and 2E, S3 Fig). Removal of N-linked glycans was confirmed using concanavalin A (con A), a lectin that binds α-D-mannose and α-D-glucose moieties found on N-linked glycans (S5C Fig). Control measurements showed that PNGase F treatment did not impact clathrin-dependent endocytosis (S5D Fig). Under our measurement conditions (200 nM protein and 12-hour incubation), neither untreated nor PNGase F–treated cells showed evidence of increased toxicity upon incubation with monomer or PFF αS_acetyl_ or αS_un_ (S5E Fig). Moreover, and strikingly, internalization of monomer and PFF αS_un_ did not demonstrate sensitivity to PNGase F treatment (Fig 2B, 2C and 2E). Lastly, we tested endoglycosidase H (Endo H), which cleaves high mannose N-linked carbohydrates, and found that this enzyme had only a minor impact on uptake of αS_acetyl_ by SH-SY5Y cells (S5B Fig).

**Fig 3:**
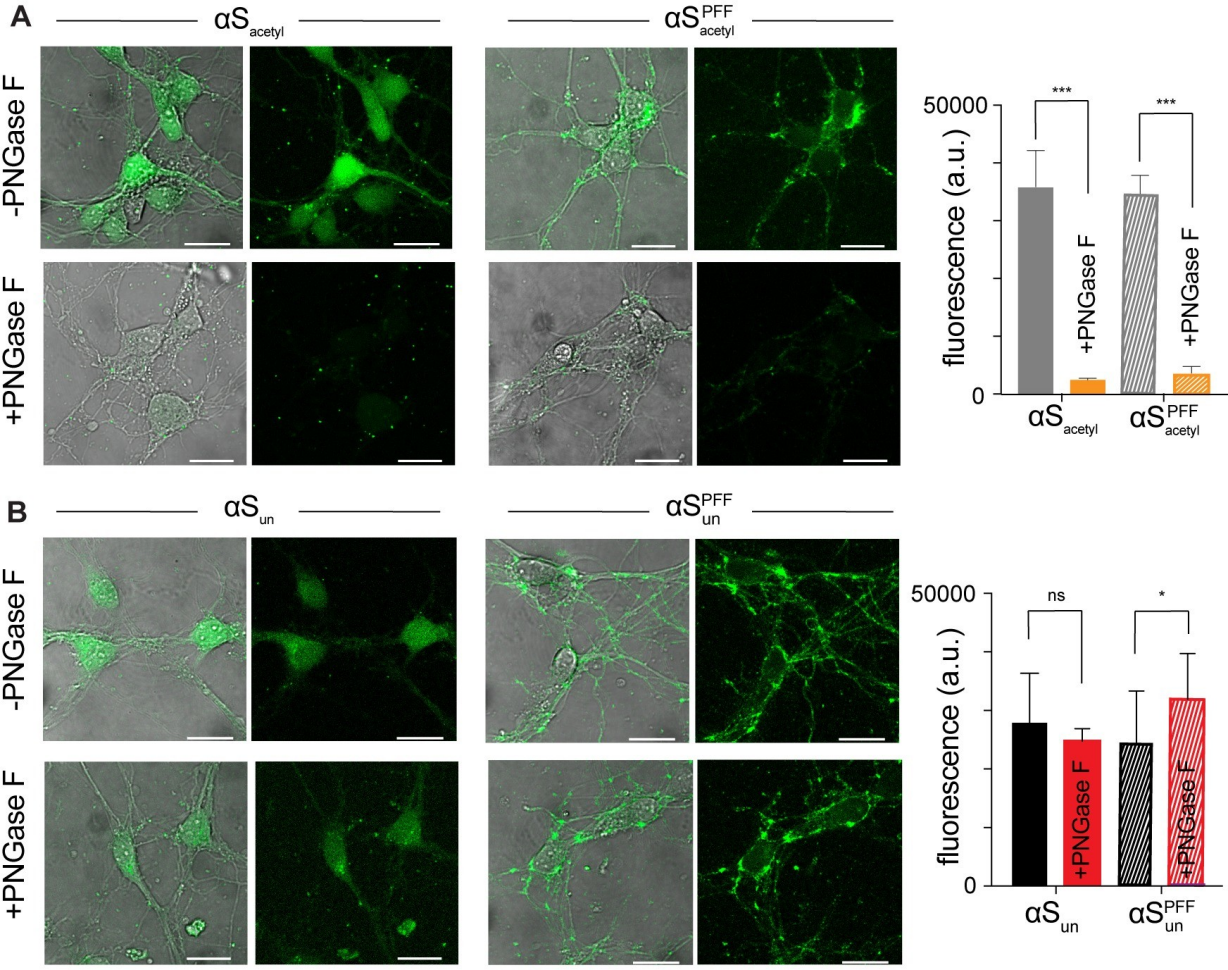
Complex N-linked glycans selectively enhance uptake of αS_acetyl_ by primary hippocampal neurons. (A) Representative images of mouse hippocampal neurons cells following 12-hour incubation with monomer or PFF αS_acetyl_-AL488, −/+ PNGase F treatment. Uptake quantified by total cellular fluorescence. (B) As in (A) but for monomer and PFF αS_un_-AL488. All internalization measurements with 200 nM monomer or 200 nM PFF (monomer units, 20:1 αS:αS-AL488); *n* = 100 cells, 3 independent experiments, *P < 0.01 and ***P < 0.0001 by Student *t* test). Scale bars = 20 μm. The underlying data for this figure can be found in S1 Data. αS, α-Synuclein; αS_acetyl_, N-terminally acetylated αS; αS_un_, unmodified αS; AL488, Alexa Fluor 488; PFF, preformed fibril; PNGase F, peptide-N-glycosidase F.

Our observations in SH-SY5Y cells were corroborated in cultured primary hippocampal neurons. Monomer and PFF αS_acetyl_ and αS_un_ were readily internalized by primary hippocampal neurons (Fig 3A and 3B). Removal of extracellular N-linked glycans by PNGase F resulted in a 10-fold decrease in the amount of internalized αS_acetyl_ (Fig 3A), while no effect was observed for αS_un_ (Fig 3B). Similar to our observation in SH-SY5Y cells, PNGase F treatment of neurons caused no defects in clathrin-dependent endocytosis (S5D Fig).

### Removal of complex N-linked glycans alters αS binding to SH-SY5Y GPMVs

**Fig 4:**
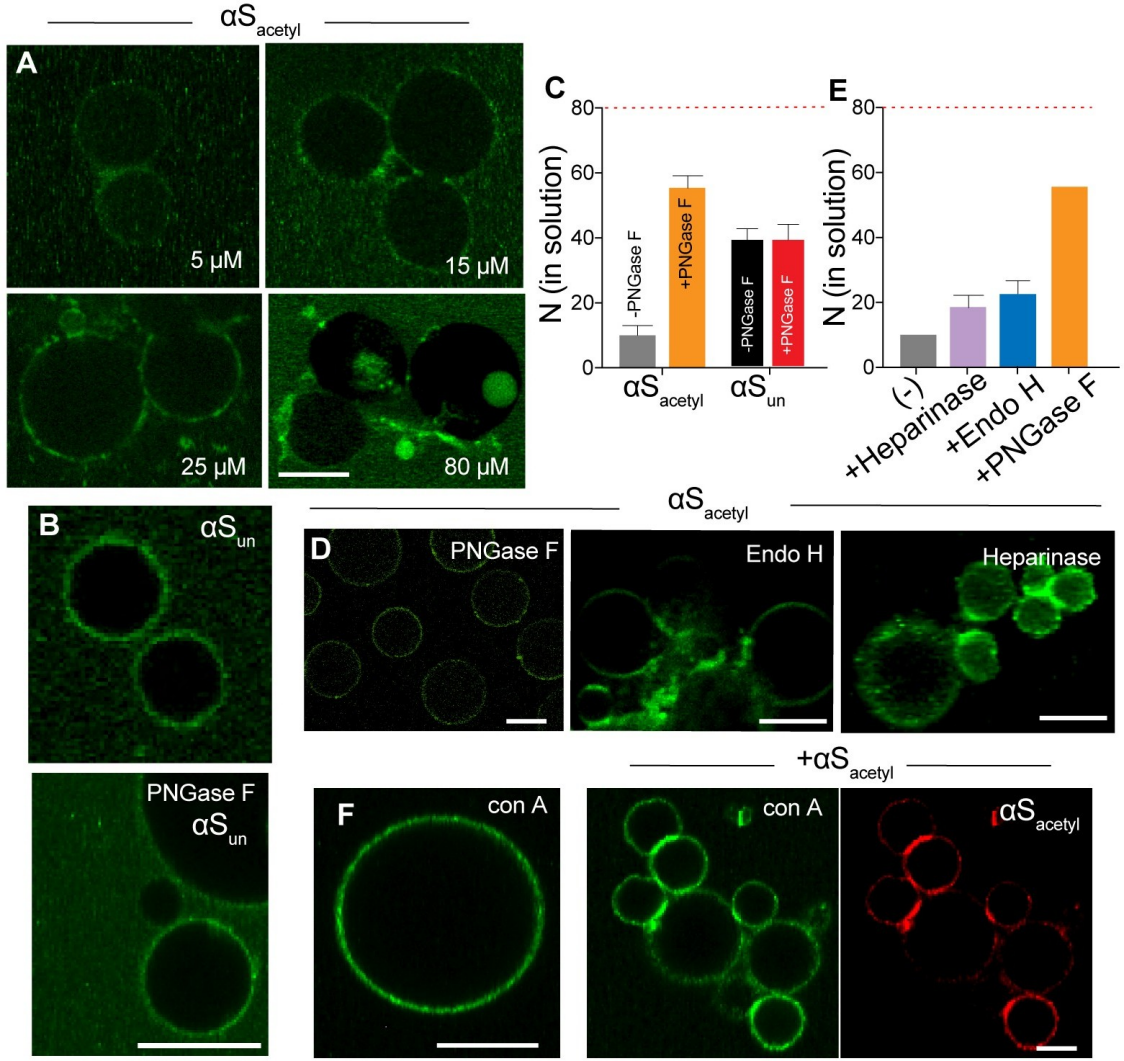
Removal of N-linked glycans disrupts binding of αS_acetyl_ to SH-SY5Y proteoliposomes. (A) Images of SH-SY5Y GPMVs incubated with 100 nM αS_acetyl_-AL488 and unlabeled αS_acetyl_ (concentrations indicated). (B) Upper: as in (A) but with 100 nM αS_un_-AL488 and 80 μM unlabeled αS_un_. Lower: as in upper panel but with treatment with PNGase F. (C) Binding of 80 nM αS_acetyl_-AL488 or αS_un_-AL488 to GPMVs quantified by FCS as loss of protein (*N*, number of molecules) from solution. The number of molecules in control wells lacking GPMVs is indicated by the red-dashed line. (D) Representative images of SH-SY5Y GPMVs incubated with 100 nM αS_acetyl_-AL488 and 80 μM unlabeled αS_acetyl_ after treatment of GPMVs with the indicated endoglycosidase. (E) Binding of αS_acetyl_-AL488 to Heparinase- and Endo H-treated SH-SY5Y GPMVs measured by FCS, as in panel C. Binding +/− PNGase F from panel C shown for comparison. (F) GPMVs incubated with 50 nM conA-AL488 in the absence and presence of 100 nM αS_acetyl_-AL594 and 80 μM of unlabeled αS_acetyl_. The data are presented as mean ± SD, *n* = 3. Scale bars = 10 μm. The underlying data for this figure can be found in S1 Data. αS, α-Synuclein; αS_acetyl_, N-terminally acetylated αS; αS_un_, unmodified αS; AL488, Alexa Fluor 488; Endo H, endoglycosidase H; FCS, fluorescence correlation spectroscopy; GPMV, giant plasma membrane vesicle; PNGase F, peptide-N-glycosidase F; SH-SY5Y, human neuroblastoma; conA-AL488, AL488-labeled conA.

Our results thus far support specific interactions between αS_acetyl_ and complex, N-linked glycans found on neurons and SH-SY5Y, but not HEK, cells that drive internalization of both monomer and PFF αS_acetyl_. To investigate the molecular details of the interactions of αS with cell-surface glycans, we used cell membrane–derived giant plasma membrane vesicles (GPMVs) that have a lipid and protein composition that closely resembles the cell plasma membrane [31]. Thus, they serve as an excellent model of the cell membrane while lacking the active processes of cells, such as uptake, allowing for binding interactions to be observed. GPMVs were harvested from SH-SY5Y cells and incubated with monomer and PFF αS_acetyl_-AL488. Both monomer and PFF αS_acetyl_-AL488 formed large, bright assemblies on the exterior of the SH-SY5Y GMPVs and caused their clustering, with larger assemblies and more clusters observed with increasing protein concentrations (Fig 4A, S6A and S6B Fig). Monomer and PFF αS_un_-AL488, on the other hand, bound more uniformly and apparently more weakly than αS_acetyl_ (Fig 4B, S6C Fig). The images present a striking contrast, suggesting differences in binding affinity for αS_acetyl_ and αS_un_. Binding of monomer αS to GPMVs was quantified by FCS (Fig 4C). In these experiments, monomer αS-AL488 was added to GPMVs in a sample chamber, and the amount of αS-AL488 that remained in solution after incubation was determined. After 60 minutes of incubation, 47% ± 4% of αS_un_ and 80% ± 6% of αS_acetyl_ were bound to the GPMVs (Fig 4C). Direct quantification of GPMV fluorescence resulting from αS-AL488 binding was consistent with the FCS results (S6D Fig).

Carbohydrates were selectively removed from the extracellular surface of the SH-SY5Y GPMVs by incubation with the same endoglycosidases used in the cell uptake measurements. Treatment with PNGase F resulted in a loss of inhomogenous binding and the bright αS_acetyl_ assemblies (Fig 4D, S6E Fig), as well as significantly decreased the amount of bound αS_acetyl_ (Fig 4E). In contrast, binding of αS_un_ to SH-SY5Y GPMVs was unaltered by PNGase F treatment (Fig 4B). By comparison, bright αS_acetyl_ assemblies were still observed after treatment with Heparinase or Endo H (Fig 4D and 4E, S6F Fig), consistent with our cellular uptake measurements. Also consistent with our cellular uptake measurements is that very weak binding was observed for αS_acetyl_ to GPMVs derived from HEK cells (S6G Fig).

Uniform binding of Alexa 488-labeled conA (conA-AL488) to SH-SY5Y GPMVs demonstrates that glycoproteins were distributed throughout the GPMV bilayer in the absence of αS_acetyl_ (Fig 4F, S6H Fig); treatment with PNGase F significantly reduced the amount of conA bound (S6H Fig). The addition of αS_acetyl_ resulted in clustering of conA-stained proteins (Fig 4F, S6I and S6J Fig). αS_acetyl_ appeared to induce assembly through binding to and noncovalently crosslinking multiple membrane glycoproteins, likely observed because GPMVs lack an intact cytoskeleton that would otherwise restrict large-scale rearrangement of plasma membrane proteins.

### αS_acetyl_ binds complex N-linked glycans with a distinct structure

**Fig 5:**
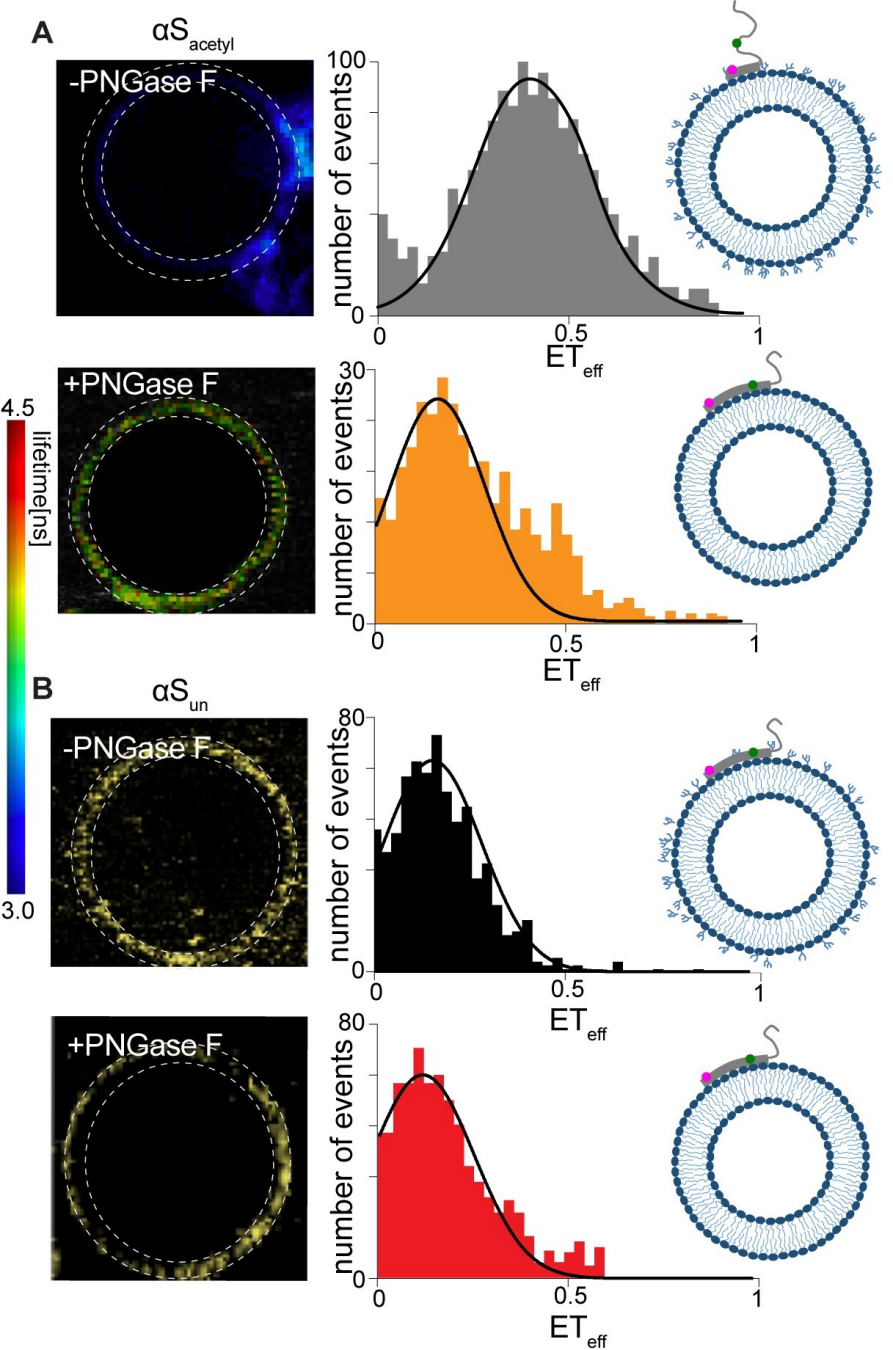
Intramolecular FLIM-FRET measurements of αS bound to SH-SY5Y GPMVs. (A) FLIM-FRET measurements of αS_acetyl_ bound to GPMVs without (upper) and with (lower) treatment with PNGase F. Left: representative image of a GPMV, colored by donor fluorophore lifetime shown in the scale bar. Right: histogram of ET_eff_ calculated from the images, with Gaussian fits shown. The pixels used to calculate the histograms are indicated by dashed lines on the GPMV images. (B) As in (A) but with αS_un._ Histograms indicated are an average of 3 GPMVs per biological replicate (*n* = 3). The underlying data for this figure can be found in S1 Data. αS, α-Synuclein; αS_acetyl_, N-terminally acetylated αS; αS_un_, unmodified αS; ET_eff_, energy transfer efficiency; FLIM, fluorescence lifetime imaging microscopy; FRET, Förster resonance energy transfer; GPMV, giant plasma membrane vesicle; PNGase F, peptide-N-glycosidase F.

Because its native function is thought to involve interactions with cellular membranes, binding of αS to synthetic lipid vesicles has been thoroughly investigated by a number of experimental methods [32–34]. The interaction is mediated through the first approximately 95 residues of αS, which form an α-helix upon binding to synthetic lipid vesicles [33,35]. To interrogate the conformational features of αS bound to GPMVs, intramolecular Förster resonance energy transfer (FRET) measurements were made. αS was labeled at residues 9 and 72, positions encompassing much of the membrane-binding domain. Mean energy transfer efficiencies (ET_eff_) of αS_acetyl_ and αS_un_ bound to SH-SY5Y GPMVs were 0.43 ± 0.08 and 0.21 ± 0.06, respectively (Fig 5A and 5B). This lower ET_eff_ measured for αS_un_ was consistent with a single, long α-helix that we have previously measured using single-molecule FRET for αS bound to synthetic lipid vesicles [35]. The higher ET_eff_ measured for αS_acetyl_ demonstrates that it binds in a distinct conformation. Strikingly, when complex N-linked glycans were removed from GPMVs by treatment with PNGase F, ET_eff_ distribution peaks of 0.19 ± 0.06 and 0.17 ± 0.03 were observed for αS_acetyl_ and αS_un_, respectively (Fig 5A and 5B)_._ Our interpretation of these findings is that, in the absence of complex N-linked glycans, αS_acetyl_ binds to GPMVs through interactions with the lipid bilayer resulting in a predominantly extended conformation. In the presence of N-linked glycans, αS_acetyl_ binding is enhanced, and it assumes a conformation distinct from the extended, membrane-bound α-helix. The higher mean ET_eff_ and relatively broad distribution of αS_acetyl_ may reflect a dynamic, disordered C-terminal half of the membrane-binding domain that does not strongly associate with the GPMVs in the presence of surface glycans. Binding of αS_un_, on the other hand, occurs primarily through its interactions with the lipid bilayer resulting in the extended helical state even in the presence of N-linked glycans. These results provide a basis for understanding why no differences in GPMV binding (Fig 4C) or in cellular uptake (Figs 2C, 2E and 3B) were observed for αS_un_ upon PNGase F treatment.

### αS_acetyl_ binds isolated N-linked glycans from SH-SY5Y cells

**Fig 6:**
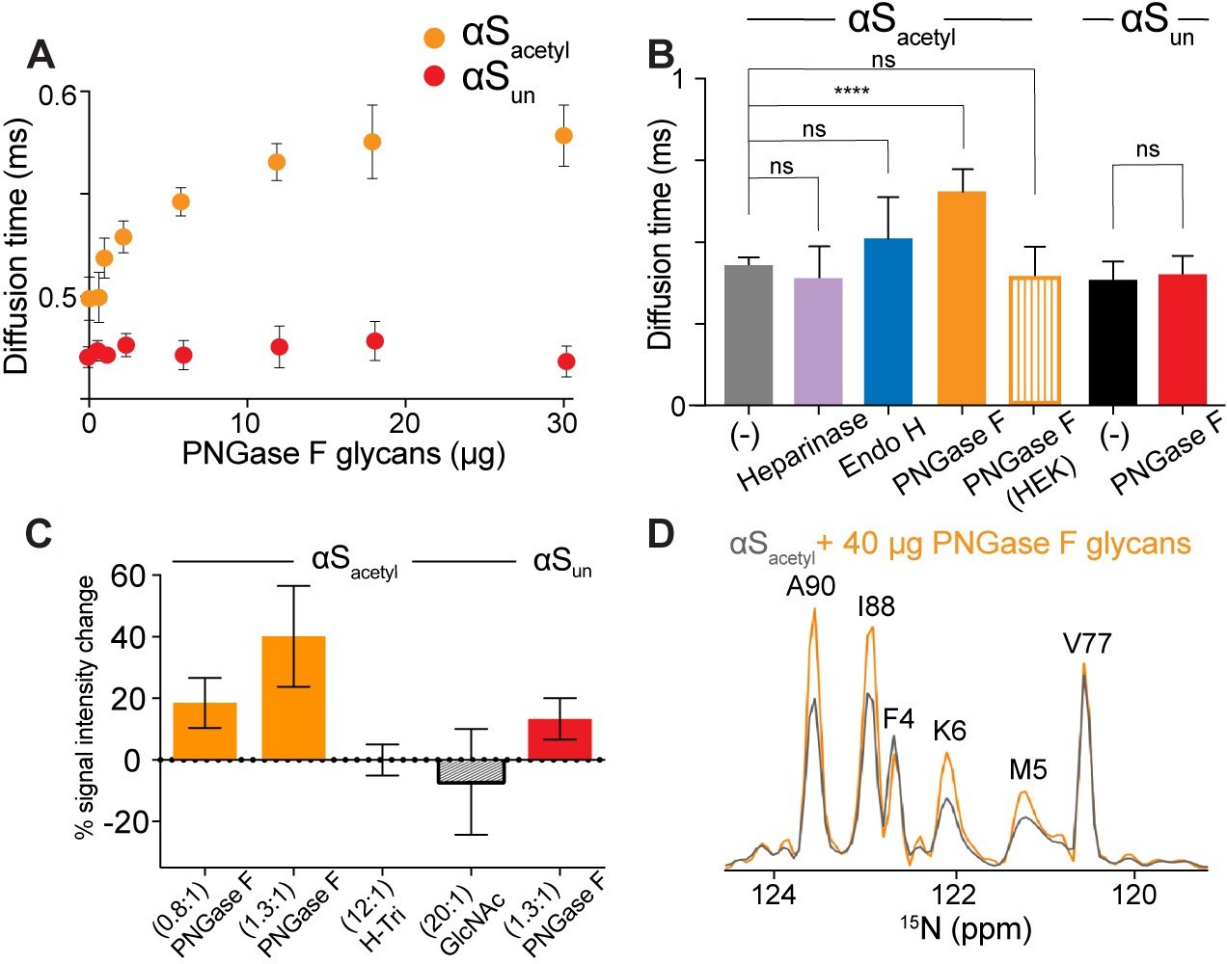
Binding of αS_acetyl_ to isolated cell-surface N-linked glycans. (A) Diffusion time of αS_acetyl_-AL488 and αS_un_-AL488 (80 nM) as a function of PNGase F–derived glycan concentration measured by FCS. (B) Comparison of αS_acetyl_-AL488 and αS_un_-AL488 binding to carbohydrates (30 μg) cleaved by the indicated endoglycosidases as measured by FCS. (C) Percent increase in peak intensity for selected residues (*n* = 9: T22, K32, T44, H50, T59, T64, G86, G93, and N103) strongly enhanced by glycan binding relative to selected residues (*n* = 9: A17, A27, L38, V40, V71, V95, I112, E130, and Y136) weakly affected by glycan binding. Measurements were performed for αS_acetyl_ in the presence of PNGase F–cleaved glycans, H-Tri, and GlcNAc and for αS_un_ with PNGase F–cleaved glycans. The ratio of sugar to protein used is indicated. (D) Cross-sections from ^15^N-^1^H HSQC spectra along the nitrogen dimension at 8.15 ppm for αS_acetyl_ in the absence (gray) and in the presence of PNGase F–derived glycans (orange). For (A–C), data are presented as mean ± SD, *n* = 3. ***P < 0.0001 by Student *t* test). The underlying data for this figure can be found in S1 Data. αS, α-Synuclein; αS_acetyl_, N-terminally acetylated αS; αS_un_, unmodified αS; AL488, Alexa Fluor 488; FCS, fluorescence correlation spectroscopy; GlcNAc, N-acetylglycosamine; H-Tri, H-Trisaccharide; ns, not significant; HSQC, heteronuclear single quantum coherence; PNGase F, peptide-N-glycosidase F.

To identify whether αS_acetyl_ binding to glycans requires either the associated glycoproteins or a lipid bilayer, binding to isolated glycans in solution was measured by FCS. SH-SY5Y cells were treated with each of the 3 endoglycosidases used in the cell and GPMV experiments, and the cleaved carbohydrates were retained. The carbohydrates were titrated into αS_acetyl_-AL488 for FCS measurements, and the autocorrelation curves (S7A Fig) were fit to extract the diffusion time and average number of fluorescent molecules, *N* (Fig 6A, S7B Fig). The diffusion time—which reflects the hydrodynamic size of the diffusing species—of αS_acetyl_ increased more than 25% with increasing concentrations of PNGase F–derived glycans, comparable to similar measurements of conA-AL488 (Fig 6A and 6B, S7C Fig). No increase in the diffusion time of αS_un_ was observed in the presence of PNGase F–derived glycans (Fig 6A). Similarly, the addition of carbohydrates obtained from Endo H or Heparinase treatment of SH-SY5Y cells or PNGase F treatment of HEK cells resulted in minimal changes in the diffusion time of αS_acetyl_ by FCS (Fig 6B, S7D Fig). Nuclear magnetic resonance (NMR) measurements showed nonuniform glycan-dependent changes in αS_acetyl_ peak intensity in the presence of PNGase F–cleaved glycans, but not simple carbohydrates (Fig 6C and 6D). These changes likely result not only from direct and specific interactions of αS_acetyl_ with the cell-derived glycans through its acetylated N-terminus, but also changes in the overall conformational ensemble as a result. Only a minimal increase in signal intensity was observed for αS_un_ with PNGase F–cleaved glycans (Fig 6C). By a filtration-based assay, both monomer and PFF αS_acetyl_ were found to pull down PNGase F–cleaved glycans from solution (S7E Fig). In contrast to GPMV images, there was no evidence of glycan-mediated assembly of αS_acetyl_ in solution (S7B Fig). This may reflect a difference in αS_acetyl_ binding to disperse glycans in solution compared to the relatively high density of glycans on the GPMV surface. Alternatively, it could be due to the absence of the relevant glycoprotein(s) because αS_acetyl_ binding may involve interactions both with N-linked glycans as well as the associated glycoprotein(s).

### Neurexin 1β drives internalization of αS_acetyl_

**Fig 7:**
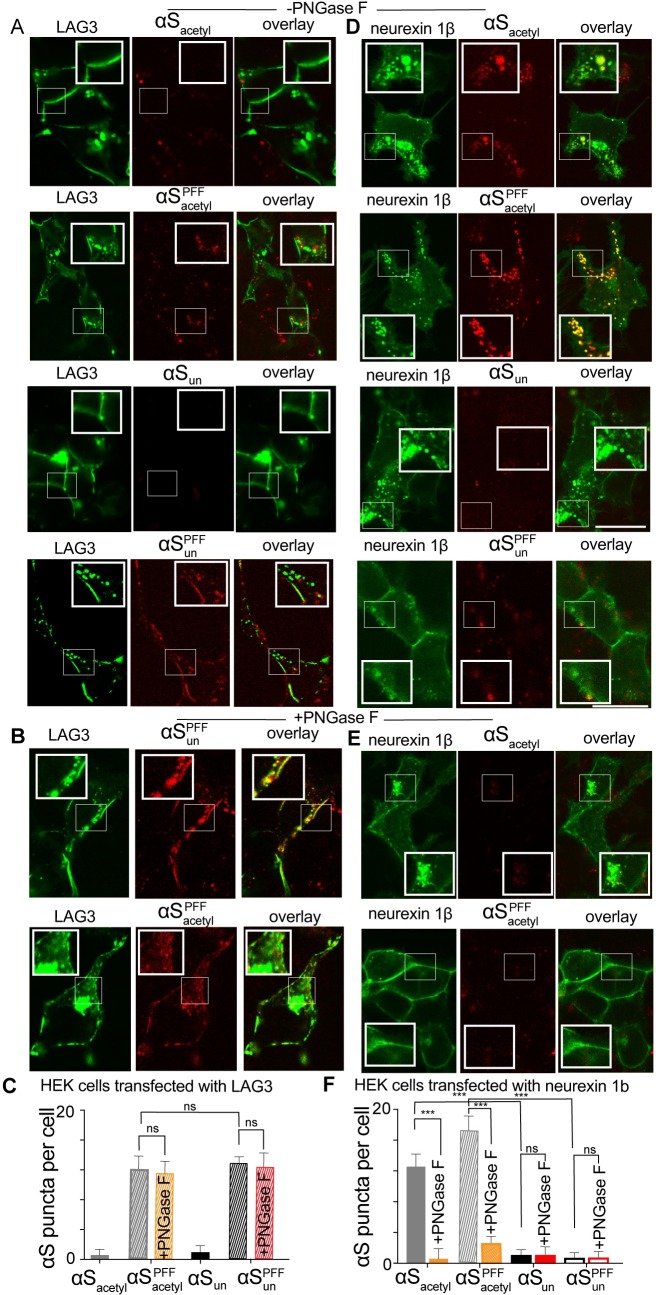
Glycosylated neurexin 1β is a receptor for αS_acetyl_. (A) HEK cells transfected with LAG3-eGFP after incubation with monomer or PFF αS_acetyl_-AL594 or αS_un_-AL594. (B) HEK cells transfected with LAG3-eGFP but treated with PNGase F prior to incubation with monomer or PFF αS_acetyl_-AL594 or PFF αS_un_-AL594. (C) Quantification of uptake of monomer and PFF αS-AL594 by LAG3-eGFP transfected HEK cells and +/− PNGase F treatment quantified by puncta analysis (for αS_acetyl_ and αS_un_ PFFs, as no significant uptake of monomer αS_acetyl_ or αS_un_ is observed without treatment). (D) As in (A) but HEK cells transfected with neurexin 1β-eGFP. (E) As in (B) but HEK cells transfected with neurexin 1β-eGFP. (F) As in (C) but HEK cells transfected with neurexin 1β-eGFP and with analysis of monomer αS_acetyl_ and αS_un_. All internalization measurements with 200 nM monomer or 200 nM PFF (monomer units, 20:1 αS:αS-AL594) *n* = 50 cells, 3 independent experiments, ***P < 0.0001 by Student *t* test). Scale bars = 20 μm. The underlying data for this figure can be found in S1 Data. αS, α-Synuclein; αS_acetyl_, N-terminally acetylated αS; αS_un_, unmodified αS; AL594, Alexa Fluor 594; eGFP, enhanced green fluorescent protein; HEK, human embryonic kidney 293T; LAG3, lymphocyte activation gene 3; PFF, preformed fibril; PNGase F, peptide-N-glycosidase F.

Our results to this point demonstrate that the uptake of both monomer and PFF αS_acetyl_ by SH-SY5Y cells or primary neurons is strongly impacted by interactions with complex, N-linked glycans. Our biophysical measurements with GPMVs and isolated carbohydrates support this observation. This prompted efforts to identify a specific glycoprotein binder partner for αS_acetyl_. One recent screen of transmembrane proteins identified neurexin 1β and lymphocyte activation gene 3 (LAG3), both of which contain a single N-linked glycosylation site in their extracellular domains, as binding partners for αS_un_ [9]. Although this study did not address the impact of N-terminal acetylation of αS, nor of glycosylation of the neurexin 1β or LAG3, it found that both receptor proteins exhibit specificity for PFF over monomer αS_un_, with the effect more striking for LAG3. To specifically address a possible role for glycosylation of these proteins in αS uptake, HEK cells were transfected with either LAG3 or neurexin 1β, each bearing an enhanced green fluorescent protein (eGFP) tag on its intracellular domain (S8A Fig). At comparable transfection levels, the proteins localized to the plasma membrane, as expected (S8B Fig). αS was labeled with AL594 (αS-AL594) to allow for simultaneous imaging of the transfected protein and exogenously added αS. In the absence of LAG3 or neurexin 1β, no uptake of αS_acetyl_ or αS_un_ monomer or PFF by HEK cells was observed (S4B Fig). Upon the addition of either αS_acetyl_ or αS_un_ PFFs to LAG3 expressing cells, colocalization with LAG3 on the plasma membrane followed by uptake was observed (Fig 7A). Consistent with the results of the screen that identified these proteins, neither colocalization nor uptake were detected for monomer αS_un_ (Fig 7A and 7C), nor did we observe it for monomer αS_acetyl_ (Fig 7A and 7C). Treatment of the LAG3-expressing cells with PNGase F did not decrease uptake of αS_un_ or αS_acetyl_ PFFs (Fig 7B and 7C).

The results from the neurexin 1β expressing HEK cells stand in striking contrast. Both monomer and PFF αS_acetyl_ were internalized by these cells where they colocalized in distinct intracellular puncta (Fig 7D and 7F). Intracellular puncta containing neurexin 1β were not observed in the absence of αS (S8A Fig), demonstrating that αS_acetyl_ drives internalization of neurexin 1β during its uptake. We saw no evidence of uptake of monomer or PFF αS_un_ in neurexin 1β expressing HEK cells (Fig 7D and 7F). At short incubation times, both αS_un_ and αS_acetyl_ PFFs colocalized with neurexin 1β on the cell surface; however, only αS_acetyl_ PFFs were internalized upon longer incubation (S8C Fig). Treatment of neurexin 1β transfected HEK cells with PNGase F greatly decreased binding and uptake of αS_acetyl_ monomer and PFF (Fig 7E and 7F). Moreover, neurexin 1β maintained its plasma membrane localization (Fig 7E). These data identify neurexin 1β and its glycosylation as key modulators of pathogenic cell-to-cell transmission of αS_acetyl_.

## Discussion

In this study, we present compelling evidence that cellular internalization of αS_acetyl_ by primary neurons and SH-SY5Y cells is dependent upon complex, N-linked glycans. αS_acetyl_ binds to these complex, N-linked glycans in solution and on cell-derived proteoliposomes. Glycan binding is a novel result for αS_acetyl_, and we propose that it is likely to be critical for recognition of functional protein binding partners. We identify one of those binding partners, neurexin 1β, and show that both its binding to αS_acetyl_ and the consequent uptake of αS_acetyl_ are dependent upon its glycosylation.

Critically, the underlying factor in our discovery is our use of αS_acetyl_ and our ability to make direct comparisons between αS_acetyl_ and αS_un._ Mass spectrometry analysis of αS derived from mammalian brain tissue and various mammalian cells types, as well as from post-mortem human brain tissue, note the presence of the N-terminal acetyl modification on both soluble and insoluble forms of the protein [11,14,15,36,37]. However, despite the fact that αS_acetyl_ can be produced in *Escherichia coli* [19], many studies—including those aimed at understanding function and uptake mechanisms of αS—still rely on the unmodified protein. Current estimates are that approximately 80% of mammalian proteins are modified by N-terminal acetylation [38]. In contrast to other protein modifications, including side-chain acetylation, no N-terminal deacetylase enzymes have yet been identified, suggesting that N-terminal acetylation is not reversible [39]. To illustrate, one recent study found fractional acetylation on lysines 6 and 10 of αS from mouse brain but that the N-terminal acetyl group was ubiquitously present [11]. For many N-terminally acetylated proteins, e.g., tropomyosin binding to actin [40], this modification is required for recognition of binding partners [41]. Our current work demonstrates that this is true for αS_acetyl_ and neurexin 1β (Fig 7) and may also be true for other cellular binding partners of αS. Moreover, the increased uptake (Figs 2 and 3), as well as the resulting enhancement of intracellular aggregate formation induced by αS_acetyl_ PFFs relative to αS_un_ PFFs (Fig 1), provide compelling evidence that N-terminal acetylation of αS has physiological consequences that thus far have been overlooked. From our findings, we speculate that the increased potency of αS_acetyl_ PFFs in inducing aggregation of endogenous neuronal αS (Fig 1) may result from more rapid internalization of αS_acetyl_ PFFs relative to αS_un_ PFFs. We anticipate that our results may help reconcile conflicting cellular and animal models with biochemical and biophysical studies of αS in which the protein may lack the appropriate modifications.

Disordered proteins such as αS_acetyl_ often participate in highly specific—but relatively low-affinity—interactions [42]. As such, multivalency provides sufficient avidity for biological interactions between disordered proteins and binding partners in vivo, in examples as diverse as tubulin polymerization [43], liquid-liquid phase separation [44], and nuclear transport [45]. Intriguingly, interactions between glycan-binding proteins—including lectins—and their binding partners are often described in the same terms. The binding between these proteins and a single glycan is often relatively low affinity (μM-mM) [46]. We estimate the apparent K_d_ for monomer αS_acetyl_ and PNGase F–derived glycans in solution from the FCS data shown in Fig 6A to be approximately 10–20 μM. Multivalency both enhances the affinity and confers specificity on the interaction, as high affinity binding only occurs when the correct cluster of glycans is present and in the correct orientation. This requirement may underlie the differences we observe for αS_acetyl_ in its interactions with SH-SY5Y and primary neurons relative to HEK cells. While complex N-linked glycans are abundant on all mammalian cells membranes, there are significant differences in the specific glycome between cell types; moreover, the glycome may be modified in response to development or disease [47]. Most glycan-binding proteins are members of well-characterized families and share similar structures or amino acid sequences [48]. To our knowledge, there are no prior examples of entirely disordered proteins showing selective binding to complex, N-linked glycans, further underscoring the novelty of our results.

Monomer αS is disordered in solution, with transient helical structure in the amino terminus [33]. N-terminal acetylation enhances the helical propensity of the first 12 residues of αS, as well as exerting long-range effects up through approximately residue 50 [49]. These changes in conformational sampling conferred by N-terminal acetylation may have a role in the selective glycan binding of αS_acetyl_ relative to αS_un_. Also relevant to our study, which finds that both monomer and PFF αS_acetyl_ interact with N-linked glycans, there are several recent structures of αS fibrils that found that the first approximately 45 residues of αS remain flexible and extended—and thus presumably available for binding—in aggregates [50–52]. That both monomer and PFF αS_acetyl_ and αS_un_ are internalized by SH-SY5Y cells, while only αS_acetyl_ uptake is impacted by N-linked glycans and only PFF αS_un_ uptake is impacted by Heparinase, strongly suggests that there are multiple modes by which αS interacts with the extracellular plasma membrane. For αS_acetyl_, binding to the extracellular membrane is driven by its specific interactions with N-linked glycans. For αS_un_, binding appears to be derived primarily from interactions with outer leaflet lipids and, for PFF αS_un_, proteoglycans. In all cases, altering the amount of bound αS, whether by cleaving a specific carbohydrate or increasing the amount of glycoprotein (as with neurexin 1β), correspondingly alters the amount of internalized αS.

One consequence of our identification of αS_acetyl_ as a glycan-binding protein is that it elicits a reconsideration of interactions between αS and putative binding partners. Many of the proteins identified as such, including glucocerebrosidase [53] and Rab3b [54], as well as the LAG3 and neurexin 1β [9] examined here, contain N-linked glycosylation sites. Consistent with the description of glycan-binding proteins above, our results with LAG3 and neurexin 1β emphasize the relevance of the specific glycans modifying the glycoprotein in determining binding of αS_acetyl_. Both LAG3 and neurexin 1β are modified by N-linked glycans; however, only the glycans on neurexin 1β mediate binding and uptake of αS_acetyl_ (Fig 7). As αS is a major target for drug development to treat Parkinson disease and other synucleinopathies, identification of αS_acetyl_ as a glycan-binding protein provides new considerations for therapeutic approaches.

## Materials and methods

### Ethics statement

The primary neurons used in this manuscript were purchased from Neurons R Us Culture Service Center at Penn Medicine Translational Neuroscience Center (PTNC) at the University of Pennsylvania. The use and care of animals were in accordance with the NIH Guide for the Care and Use of Experimental Animals, and protocols were approved by the University of Pennsylvania Institutional Animal Care and Use Committee (IACUC).

### αS expression and purification

αS was expressed in *E*. *coli* BL21 cells; for αS_acetyl_, BL21 stocks containing the N-terminal acetyltransferase B (NatB) plasmid with orthogonal antibiotic resistance were used. The purification of both αS_acetyl_ and αS_un_ protein was carried out as previously described [19], with minor modifications. Briefly, two ammonium sulfate cuts were used (0.116 g/mL and 0.244 g/mL) with αS precipitating in the second step. The pellet was resolubilized in Buffer A (25 mM Tris [pH 8.0], 20 mM NaCl, 1 mM EDTA) with 1 mM PMSF and dialyzed against Buffer A to remove ammonium sulfate. Dialyzed samples were loaded to an anion exchange column (GE HiTrap Q HP, 5 ml) and eluted with a gradient to 1 M NaCl. αS elutes at approximately 300 mM NaCl. Fractions containing αS were pooled and concentrated using Amicon Ultra concentrators (3,000 Da MWCO). Concentrated samples were then loaded to a size exclusion column (GE HiLoad 16/600 Superdex75) and eluted at 0.5 ml/minute. Fractions containing αS were again pooled and concentrated, then stored at −80°C. All αS constructs used in this work were checked by matrix-assisted laser desorption/ionization (MALDI) to confirm correct mass and presence of acetylation (S1 Fig).

For NMR measurements, 15N-labeled αS was grown in *E*. *coli* BL21 stocks containing the NatB plasmid in minimal medium (6 g/L Na_2_HPO_4_.7H_2_O, 3 g/L KH_2_PO_4_, 0.5 g/L NaCl, 1 mM MgS0_4_, 300 μM CaCl_2_, 0.5 g/L ^15^NH_4_Cl) instead of LB medium and purified as described above.

### αS labeling

αS was site-specifically labeled at a single position by introduction of a cysteine at either residue 9 or residue 130. For labeling reactions, freshly purified αS (typically 200–300 μL of approximately 200 μM protein) was incubated with 1 mM DTT for 30 minutes at room temperature to reduce the cysteine. The protein solution was passed over 2 coupled HiTrap Desalting Columns (GE Life Sciences, Pittsburgh, PA) to remove DTT and buffer exchanged into 20 mM Tris (pH 7.4), 50 mM NaCl, and 6 M guanidine hydrochloride (GdmCl). The protein was incubated overnight at 4°C with stirring with 4× molar excess AL488 or AL594 maleimide (Invitrogen). The labeled protein was concentrated and buffer exchanged into 20 mM Tris (pH 7.4), 50 mM NaCl using an Amicon Ultra 3K Concentrator (Millipore, Burlington, MA), with final removal of unreacted dye and remaining GdmCl by passing again over a set of coupled desalting columns equilibrated with 20 mM Tris (pH 7.4), 50 mM NaCl.

For dual fluorophore labeling for FRET measurements, cysteines were introduced at residues 9 and 72. The protein was labeled as described above, with the following modifications. The protein was first incubated with donor fluorophore AL488 maleimide at a ratio of protein:dye of 1:0.5 for 2 hours at room temperature with stirring. Then, 4× molar excess of acceptor fluorophore AL594 maleimide was added, and the reaction was continued overnight at 4°C. The labeled protein was separated from unreacted dye as described above. αS labeled at these positions has been extensively studied in our lab; as documented in our previous publications, they do not perturb αS binding to lipid membranes and serve as excellent reporters of different conformations of membrane-associated αS [35,55].

### Fibril formation

αS PFFs were prepared as previously described [21]. Briefly, 100 μM αS was mixed with 5 μM αS-AL488 in 20 mM Tris (pH 7.4) and 100 mM NaCl. To induce aggregation, this solution was incubated at 37°C for 5 days with agitation (1,500 rpm on an IKA MS3 digital orbital shaker) in parafilm-sealed 1.5 mL Eppendorf tubes to ensure minimal solvent evaporation. The aggregation reaction was analyzed by Congo Red absorbance by diluting 10 μl of the aggregation solution in 140 μl 20 μM Congo Red. The mature fibrils were then pelleted by centrifugation (13,200 rpm for 90 minutes at 4°C), and the supernatant was removed. Fibers were resuspended in an equal volume (relative to supernatant) of 20 mM Tris (pH 7.4), 100 mM NaCl. Mature fibers were subsequently fragmented on ice using a sonicator (Diagenode UCD-300 bath sonicator) set to high, 30 seconds’ sonication followed by a delay period of 30 seconds—10 minutes total—to form PFFs.

### Assessment of fibrillar material

TEM and PAGE were used to characterize fibrillar αS (S1 Fig). For TEM, 10 μL of aggregated protein samples (from both before and after sonication) were incubated on TEM grids (400-mesh Formvar carbon-coated copper, Electron Microscopy Sciences, Hatfield, PA) for 1 to 2 minutes. Sample solution was wicked with filter paper, and the grid was washed with water to remove any excess material and improve background contrast. Grids were then incubated with 1% (w/v) aqueous uranyl acetate solution (10 μL) for 30 to 60 seconds. Excess uranyl acetate was wicked away with filter paper, and grids were air dried. TEM images were collected using a JOEL JEM 1011 TEM (JEOL, Peabody, MA) (operating voltage 100 kV) equipped with a charge-coupled device camera (ORIUS 832. 10W; Gatan, Pleasanton, CA). The lengths of the PFFs post sonication were quantified using the Fiji measuring tool on TEM images [57].

For PAGE, aggregated protein solutions were centrifuged to pellet the aggregated material. The supernatant was removed and pellet was resuspended in the starting volume of buffer. Both supernatant and resuspended pellet (20 μL) were loaded on a 4% to 12% polyacrylamide gel. Gels were imaged using a Typhoon FLA7000 gel imager (GE Life Sciences, Pittsburgh, PA) using Coomassie stain mode.

### Cell culture

SH-SY5Y and HEK cells were grown at 37°C under a humidified atmosphere of 5% CO_2_. The SH-SY5Y cells were cultured in Dulbecco's Modified Eagle's Medium (DMEM) plus 10% fetal bovine serum, 50 U/ml penicillin, and 50 μg/ml streptomycin. The HEK cells were cultured in DMEM supplemented with 10% FBS, 2 mM L-glutamine, and 100 units/ml penicillin-streptomycin.

Cells were passaged upon reaching approximately 95% confluence (0.05% Trypsin-EDTA, Life Technologies, Carlsbad, CA), propagated, and/or used in experiments. Cells used in experiments were pelleted and resuspended in fresh media lacking Trypsin-EDTA.

### Primary neuronal culture

Primary neuronal cultures were obtained from the Neurons-R-Us facility at the University of Pennsylvania. They were prepared from E15-E17 embryos of CD1 mice. All procedures were performed according to the NIH Guide for the Care and Use of Experimental Animals and were approved by the University of Pennsylvania IACUC. Dissociated hippocampal neurons were plated onto sterile, poly-D-lysine coated on IBIDI chambers at 200,000 cells/coverslip for live cell imaging and were allowed to mature for 5 days in complete neuronal medium (neurobasal without phenol red [Thermo Fisher, Waltham, MA], 5% B27 supplement [Thermo Fisher]). Medium was partially exchanged every 3 to 4 days.

### GPMVs

GPMVs are blebs obtained directly from the cell plasma membrane that contain lipid bilayers and the embedded membrane proteins but lack the other biological components of the cell [31]. GPMVs were isolated from SH-SY5Y and HEK cells according to established methods [56]. Briefly, cells were plated in 25 cm^2^ culture flasks and cultured for 48 hours, washed with GPMV buffer (10 mM HEPES, 150 mM NaCl, 2 mM CaCl_2_ [pH 7.4]) twice, and then exposed to 25 mM formaldehyde and 2 mM DTT for 2 hours to induce blebbing. To reduce the content of DTT, GPMVs were dialyzed in GPMV buffer prior to use in experiments. GPMVs were also created using *N*-ethylmaleimide as the blebbing reagent, with comparable results. The phospholipid content of final material was measured by total phosphate assay.

### Phosphate assay

Lipid concentrations for GPMV preparations were estimated by measuring total phosphate, assuming that all measured phosphate is from phospholipids and that all lipids are phospholipids. This is a practical assumption designed to ensure reproducibility.

### Enzymatic cleavage of carbohydrates

For cleavage of carbohydrates from GPMVs, endoglycosidases were added to the GPMVs in GPMV buffer at concentrations recommended by the manufacturers (PNGase F: 5,000 units/ml; Endo H: 2,500 units; Heparinase I/III: 2,500 units) and incubated at 37°C for 6 hours. PNGase F was tagged with a chitin binding domain (Remove-IT PNGase F, New England Biolabs, Ipswich, MA). For the images shown in this manuscript, the enzyme was removed by incubation of GPMVs with 50 μl of chitin binding magnetic beads. Control experiments were conducted without removal of PNGase F and found to be comparable. Cleavage of N-linked glycans by PNGase F was confirmed by comparing images of GPMVs +/− PNGase F treatment after incubation with 50 nM conA, a lectin that binds to binding to α-D-mannose and α-D-glucose moieties, or wheat germ agglutinin, a lectin that binds to N-acetyl-D-glucosamine and sialic acid. A significant decrease in the amount of both proteins is observed in these images (S6B Fig).

For cleavage of carbohydrates from cells, cells were first plated for 42 hours. After 42 hours, media were removed from cells and replaced with FBS-free media complimented with the endoglycosidase (PNGase F: 5,000 units/ml; Endo H: 2,500 units; Heparinase I/II: 2,500 units). The cells were incubated at 37°C under a humidified atmosphere of 5% CO_2_ for an additional 6 hours. The media were then removed and replaced with cell growth media prior to the addition of αS. Cleavage of N-linked glycans from cells was confirmed by comparing images +/−- PNGase F treatment after incubation with 50 nM conA-AL488, showing a significant reduction in the amount of conA bound (S5D Fig).

To ensure that PNGase F is removed from GPMVs or cells after incubation (and therefore does not remain associated with either, blocking potential αS binding sites), we compared the amount of PNGase F added to either GPMVs or cells with that removed after incubation. PNGase F (5,000 units/ml) containing a chitin domain was added to chambers containing either GPMVs or cells and was incubated at 37°C for 6 hours, as for the experiments described above. After incubation, the buffer or media containing PNGase F were removed and incubated with chitin magnetic beads to isolate and concentrate the enzyme. Blank chambers containing only PNGase F in buffer or media were subjected to the same treatment; 20 μL of each sample was run on a 4%–12% polyacrylamide gels and stained with Coomassie blue. Gels were imaged using a Typhoon FLA7000 gel imager (GE Life Sciences, Pittsburgh, PA) using Coomassie stain mode. The gels indicate that essentially all of the enzyme is removed (S9A Fig).

### Quantification of carbohydrates

Concentrations of carbohydrates isolated from GPMVs or cells were quantified by using the Total Carbohydrate Quantification Assay Kit (Abcam, Cambridge, MA) following the manufacturer’s instructions. Briefly, the carbohydrates are first hydrolyzed to monomer sugar units and then converted to furfural or hydrofurfural. These compounds are converted to chromogens, which can be detected by absorbance at 490 nm. Glucose was used to generate a standard curve for calculation of the total carbohydrate concentration of the samples.

### αS-captured carbohydrate pull-down assay

Carbohydrates cleaved and isolated from GPMVs (50 μg) were incubated with 100μM αS in 100μL 10 mM HEPES, 150 mM NaCl, 2 mM CaCl_2_ (pH 7.4) for 1 hour at room temperature. Binding reaction mixes were transferred to Amicon Ultra (3,000 Da MWCO) centrifugal concentration devices that had been washed with 500μL deionized water. The concentrators were centrifuged for 5 minutes at 4,200 rpm and the filtrates collected. The flow-through contains carbohydrates that did not bind αS and thus were not retained in the chamber of the concentrator. The amount of carbohydrate in the flow-through was quantified by the total carbohydrate assay as described above. The fraction of carbohydrate bound and retained by αS (*C_captured_*) was calculated relative to the starting concentration (50 μg) of the carbohydrate mixture, using

$$C_{captured}=C_{total}-C_{flow-through}$$ (1)

where *C_total_* corresponds to the absorption of the starting stock concentration of carbohydrates used and *C*_*flow−through*_ is the absorption of the flow-through.

Samples containing αS with no glycans or conA with 50 μg glycans were used as negative and positive controls, respectively. As expected, no signal at 490 nm was detected for the αS-only sample; the conA results are shown in S7E Fig. The absorbance of flow-through at 280 nm was measured, confirming that all protein was retained in the concentrator and could not be detected in the flow-through.

### Cell imaging and analysis

All cell imaging was carried out by confocal fluorescence microscopy using an Olympus FV3000 scanning system configured on a IX83 inverted microscope platform with a 60× Plan-Apo/1.1-NA water-immersion objective with DIC capability (Olympus, Tokyo, Japan). For all experiments, the gain setting for the blue channel was kept constant from sample to sample (excitation 488 nm, emission BP 500–540 nm). For detection of αS-AL594, the green channel was used (excitation 561 nm, emission BP 570–620 nm). Images were obtained in 8-well ibidi chambers (μ-Slide, 8-well glass bottom, ibidi GmbH, Germany) coated with Poly-D-lysine and were seeded with 20,000–25,000 cells/well. Cells were cultured for 48 hours after passage before beginning experiments. For cellular uptake experiments, 200 nM αS-AL488 was incubated with cells for 0 to 24 hours before acquiring images. For experiments using deglycosylated cells, cells were pretreated with the tested endoglycosidase for 6 hours as described above prior to addition of protein. For colocalization with lysosomes, cells were treated with 75 nM Lysotracker Deep Red (Life Technologies, Carlsbad, CA) for 1 hour prior to imaging. For all experiments, the gain setting for each channel was kept constant from sample to sample.

Image acquisition and processing were performed with the software accompanying the FV3000 microscope and Image J software [57]. For SH-SY5Y cells, internalized αS was quantified either by analysis of the punctate structures in the cells or by the total cellular fluorescence; for primary neurons, internalized αS was quantified by total cellular fluorescence. For total cellular fluorescence, the integrated fluorescence intensity of the cells is reported. Cellular puncta were analyzed using the Image J particle analysis plug-in. This algorithm detects puncta through a user-defined threshold and counts the number of puncta that meet or exceed the threshold. The threshold was initially defined by manual identification and averaging of a subset of puncta. Colocalization with lysosomes was computed by obtaining a Pearson coefficient using the ImageJ plugin for colocalization (Coloc_2).

### Endocytosis inhibition

For inhibition of exocytosis experiments, monomer αS-AL488 (200 nM) or PFFs (200 nM in monomer units, 20:1 αS:αS-AL488) was initially incubated with cells for 30 minutes at 4°C. Control cells were moved to 37°C while the endocytosis-inhibited cells were incubated at 4°C for another 4 hours before acquiring images. For all experiments, the gain setting for each channel was kept constant from sample to sample. Image acquisition and processing were achieved using the software accompanying the FV3000 microscope and Image J software [57].

### GPMV imaging and analysis

All GPMV images were carried using a PicoQuant MicroTime 200 time-resolved fluorescence system based on an inverted Olympus IX73 microscope (Olympus, Tokyo, Japan) with a 60× Plan-Apo/1.4-NA water-immersion objective using a 482 nm excitation laser and a frame size of 512 × 512 pixels. Images acquired with this instrument were in lifetime mode but were integrated to obtain intensity-based images comparable to typical confocal images. This instrument has the advantage of very sensitive avalanche photodiode detectors (SPADs) that are capable at detecting nM concentrations of protein. Fluorescence intensities were analyzed via the lifetime mode (both intensity and FRET images) using SymPhoTime 64 (PicoQuant, Berlin, Germany). The intensity of images was then adjusted on ImageJ analysis program [57].

For FLIM-FRET experiments, measurements were made of donor-only and donor-acceptor labeled proteins. SPAD signals were processed with the TimeHarp 300 photon counting board and analyzed with the SymPhoTime 64 software (PicoQuant, Berlin, Germany) taking into account the instrument response function to allow consideration of short lifetime components with a high accuracy. FLIM images were acquired for 180 seconds, with a pixel integration time of 40 μs per pixel and an average photon count rate of 10,000–30,000 counts per second. Regions of interest of the GPMV membrane were selected from FLIM images, and fluorescent lifetimes were obtained from TCSPC decay curves fitted by an exponential equation using the SymPhoTime 64 software. Fitting of the fluorescence images was then performed pixel wise with a single exponential model on all pixels above an intensity threshold of 200 photons. By characterizing donor lifetime in the absence and presence of acceptor, ET_eff_ can be calculated from:

$${ET}_{eff}=1-\frac{\tau_{DA}}{\tau_{D}}$$ (2)

where τ_DA_ and τ_D_ and are the donor excited state lifetime in the presence and absence of acceptor. Six FLIM images were recorded for each of 3 biological repeats per condition. The histograms shown in Fig 5 represent ET_eff_ values for selected pixels from equatorial sections of the GPMVs as indicated in the images to their left. The histograms were fit with a Gaussian function to extract the mean ET_eff_.

Images were obtained in 8-well NUNC chambers (Thermo Scientific, Rochester, NY) containing 250 μl of GMPV at 5 μM phospholipid concentration and 80 nM of αS-AL488. For all experiments using these chambers, the chambers were passivated by polylysine-conjugated PEG treatment to prevent any nonspecific absorption to the chamber surfaces [55]. Quantification of fluorescence on GPMVs resulting from bound αS-AL488 was calculated by determining the integrated intensity per pixel. The fluorescence intensities of 10 GPMVs per condition were quantified.

### FCS

FCS measurements were carried out on a lab-built instrument, as described previously [35]. A 488-nm–wavelength laser was adjusted to 5 μW prior to entering the microscope. Fluorescence emission was collected through the objective and separated from laser excitation using a Z488RDC Long-Pass Dichroic and an HQ600/200M Band-Pass Filter (Chroma, Bellows Falls, VT). The fluorescence emission was focused into the aperture of a 50-μm–diameter optical aperture fiber (OzOptics, Canada) directly coupled to an avalanche photodiode. A digital correlator (Flex03LQ-12; Correlator.com) was used to generate the autocorrelation curves. For each experiment, 30 autocorrelation curves of 10 seconds each were acquired and averaged together to obtain statistical variations. These average autocorrelation curves were then fit to a function for single fluorescent species undergoing Brownian motion in a three-dimensional Gaussian volume weighted by the inverse square of the SD:

$$G(\tau )=\frac{1}{N}\times \frac{1}{1+\frac{\tau }{\tau_{D}}}\times \sqrt{\frac{1}{1+\frac{s^{2}\tau }{\tau_{D}}}}$$ (3)

where G(τ) is the autocorrelation function for translational diffusion as a function of time τ, *N* is the average number of fluorescent molecules in the laser focal volume, and τ_D_ is the translational diffusion time of the particles. The structure factor, s, is the ratio of the radial to axial dimensions of the focal volume and was obtained from a calibration procedure using AL488 hydrazide dye solutions (s = 0.2) and fixed for all subsequent fitting. Several datasets of autocorrelation curves obtained in the presence of carbohydrates were analyzed considering a single component system. For carbohydrate binding, both the diffusion time, τ_D_ (Fig 6A and 6B, S4C, S7A, S7C, S7D and S9B Figs), and the number of molecules, *N* (Fig 4C and 4E, S7B Fig), were analyzed.

### GPMV binding and cellular uptake by FCS

FCS was used to monitor αS both binding to GPMVs and uptake by cells. FCS measurements were made on the instrument described above. For GPMV binding, experiments were performed in 8-well NUNC chambers (Thermo Scientific, Rochester, NY) containing 250 μl of GMPV at 5 μM phospholipid concentration. The laser focal volume was located to a height of 100 μm from the bottom surface of the wells, above all GMPVs. αS-AL488 (80 nM) was added to wells with GPMVs, and the autocorrelation measurements were taken immediately and after 60 minutes. Each curve was integrated for 30 seconds and repeated 10 times, and data were analyzed as described above. Binding was quantified by measuring the change in the number of molecules, *N*, of fluorescently labeled αS present in the solution surrounding the GMPVs relative to the starting concentration. There was no change in the diffusion time of the αS that remained in the cell media over this time course, evidence that protein is stable and the fluorophore intact (S9B Fig).

For cell uptake experiments, cells were plated in phenol red-free medium in 8-well ibidi chambers (μ-Slide, 8-well glass bottom, ibidi GmbH, Germany) coated with Poly-D-lysine wells and cultured for 48 hours prior to measurements. Control wells contained the same volume of media without cells; these control for nonspecific adsorption of protein to the well surfaces. αS-AL488 (200 nM) was added to the wells at the start of the experiment, and the autocorrelation curves were taken in the medium well above the cells, at a height of 100 μm from the bottom surface of the wells. The curves were collected at regular intervals, over a period ranging from 2 to 24 hours, and each autocorrelation curve integrated for 30 seconds, repeated 10 times. Data were analyzed as described above. Cellular uptake was assessed by measuring the change in the number of molecules of fluorescently labeled αS present in the media surrounding the cells relative to the starting concentration. In between measurements, the chambers were returned to the incubators to maintain the temperature in the wells at 37°C. As a negative control for both the GPMV binding and cell uptake studies, GPMVs or cells were incubated with 80 nM eGFP. There was no evidence of eGFP binding to the GPMVs nor of internalization by SH-SY5Y cells (S9C Fig).

### Cellular uptake by PAGE

As an orthogonal approach to the FCS and imaging approached described above, uptake of both monomer and PFF αS were quantified by PAGE. SH-SY5Y cells were incubated with 200 nM αS, as described above for FCS uptake experiments. After incubation for the desired time (1, 3, 5, 8, 12, or 24 hours), the media were removed from cells and stored at 4°C. After all samples were collected, 20 μl of each sample was run on a 4%–12% polyacrylamide gel.

To quantify the amount of protein internalized, an identical set of experiments was carried out, with modifications as described following. For each time point, transferrin-AL488 (100 nM) was added 30 minutes prior to the due time of the time point to serve as a loading control. At the desired time points, the cells were detached from the wells by 0.05% Trypsin-EDTA (Life Technologies Carlsbad, CA), pelleted, and lysed in 250 μl RIPA lysis buffer (Thermo Fisher, Waltham, MA). Cell lysates (20 μl of stock) were run on PAGE gels. Cell lysates (20 μl) were run on 4%–12% polyacrylamide gels.

Gels of both extracellular and internalized αS were imaged using a Typhoon FLA7000 gel imager (GE Life Sciences, Pittsburgh, PA) using fluorescent imaging mode to detect αS-AL488 or transferrin-AL488. Image J was used to quantify the bands [57].

### Statistical analysis

Data are expressed as the mean±SD and were examined by a one-way analysis of variance (*n* = 3). More than 3 experiments were performed, and similar results were obtained. *P* < 0.05 was considered to be significant.

### Trypan blue quenching

Trypan blue solution 0.4% (Thermo Fisher, Waltham, MA) and fresh neurobasal without phenol red, B27, or antibiotic supplementation were equilibrated at 37°C. A 10× dilution of trypan blue was prepared freshly in the warmed neurobasal media. The trypan blue solution was then added to cells dropwise and incubated at 37°C for an hour prior to imaging. Trypan Blue quenching was performed for all imaging experiments performed using PFFs and when monomer protein was introduced in experiments utilizing primary neurons. Trypan Blue quenching was used to eliminate signal from extracellular material, allowing the fluorescence quantification of intracellular fluorescence signal.

### Propagation of amyloid in primary neurons

Primary wild-type mouse hippocampal neurons (obtained as described above) were grown for 6 days on round coverslips prior to addition of αS_acetyl_ or αS_un_ PFFs (100 nM final PFF concentration). Cells were fixed with 4% (wt/vol) paraformaldehyde and costained with antibodies specific to αS phosphorylated at serine 129 (rabbit monoclonal phospho 129, 1:250 dilution) and neuronal tubulin (mouse monoclonal anti-β-III tubulin, 1:100 dilution) after 3-, 7-, and 10-day incubations with PFFs. Primary antibodies were visualized by secondary staining with AL488 donkey anti-rabbit IgG (Invitrogen, Carlsbad, CA) and AL647 goat anti-mouse IgG (Invitrogen, Carlsbad, CA) (1:1,000 dilution).

### Transfection of HEK cells

HEK cells were transfected with plasmid encoding LAG3-eGFP or Neurexin 1 β-eGFP by Lipofectamine 3000, following the manufacturer’s directions. The media were removed from cells 48 hours after transfection and replaced with FBS-free media complimented with PNGaseF (5,000 units/ml) for experiments that required PNGaseF treatment. The cells were then incubated at 37°C under a humidified atmosphere of 5% CO_2_ for an additional 6 hours. The media were then removed and replaced with cell growth media prior to the addition of αS. Cells were incubated with monomer αS-AL594 (final concentration 200 nM) or PFF αS (final concentration 200 nM monomer units, 1:20 labeled:unlabeled) for either 1 hour or 12 hours prior to imaging.

### Flow cytometry

To quantify LAG3-eGFP and Neurexin 1 β-eGFP expression levels, HEK cells were plated and transfected as above. After 48 hours, cells were detached using 0.05% Trypsin-EDTA, centrifuged, and washed with PBS + 2mM EDTA and 2% BSA. The cells were then treated with the Zombie Yellow fixable cell viability kit (BioLegend San Diego, CA) at room temperature in PBS + 2 mM EDTA for 20 minutes and then fixed at 4°C using Cytofix for 20 minutes. Cells were then placed in PBS + 2 mM EDTA and 2% BSA. Data were collected on an LSR II flow cytometer (BD Biosciences) and postcollection data were analyzed using FlowJo (Treestar, flowjo.com) (S8B Fig). Gating was performed on live single cells in the lipofectamine-only control.

### Cell viability

Cell viability was measured colorimetrically using the Cell-Titer Blue (CTB, Promega, Madison, WI) fluorescence-based assay. Cells were plated at a density 5,000 cells/well in 96-well plates (BD Biosciences, San Diego, CA). Protein was directly introduced to each well after 48 hours of culture and then incubated for an additional 48 hours. After incubation, 30μL CTB reagent was added to each well and incubated at 37°C and 5% CO_2_ for 2.5 to 5 hours. Fluorescence of the resorufin product was measured on a FluoDia T70 fluorescence plate reader (Photon Technology International, Birmingham, NJ). Wells that included vehicle but not protein served as the negative control (0% toxic), and wells containing 10% DMSO were the positive control (100% toxic). Percent toxicity was calculated using the following equation:

$$\%Toxicity=100-\left[100(\frac{\langle S\rangle -\langle P\rangle }{\langle N\rangle -\langle P)})\right]$$ (4)

Each independent variable is the average of 8 plate replicates from the negative control (<*N>*), positive control (<*P*>), and samples (<*S*>). Results presented for viability experiments are an average of 3 such experiments conducted independently on different days. Error bars represent the standard error of the mean.

### NMR

^1^H-^15^N HSQC NMR titrations were carried out at 25°C using Varian 600 MHz or Agilent 800 MHz spectrometers equipped with room temperature probes. A uniformly labeled ^15^N-αS solution was added to either N-linked glycans obtained by PNGase F cleavage from SH-SY5Y cells or commercially available mono- or trisaccharides at the concentrations indicated in Fig 6C in GPMV buffer (10 mM HEPES, 150 mM NaCl, 2 mM CaCl_2_ [pH 7.4], 10% D_2_O; final concentration of ^15^N-αS was 350 μM). HSQC spectra were collected with VnmrJ software (openvnmrj.org) using built-in pulse sequence including WATERGATE solvent suppression and were analyzed with Mnova software suite (Mestrelab; mestrelab.com/software/mnova/). Standard parameters for zero-filling, apodization, and baseline correction were applied. ^1^H chemical shifts were referenced using water resonance, and ^15^N chemical shifts were referenced indirectly based on gyromagnetic ratios of respective nuclei. Previously assigned αS backbone resonances were used [49].

The PNGase F–derived glycans are a heterogeneous mixture of complex glycans of various sizes and monosaccharide building blocks. For comparison with the mono- and trisaccharide measurements, we approximated the amount of PNGase F–derived carbohydrate from μg (as determined by the carbohydrate quantification assay described above) to μM using the molecular weight of glucose, 180.16 g/mol. Thus, the 1.3:1 molar ratio reported in the NMR refers to the approximate number of available monosaccharide groups from the PNGase F glycans; the actual concentration of complex N-linked glycans is much lower.

Binding to the PNGase F–derived glycans results in nonuniform peak intensity increases throughout the sequence of αS. For analysis, 9 peaks showing large increases (T22, K32, T44, H50, T59, T64, G86, G93, and N103) and 9 peaks showing small or no increases (A17, A27, L38, V40, V71, V95, I112, E130, and Y136) were selected to roughly cover the entire sequence. The same residues were used for each analyzed dataset. For each set of peaks, the relative magnitude of increase (compared to αS in solution without glycans) expressed as a percentage was calculated and averaged.

## Supporting information

S1 Data Excel spreadsheet containing, in separate sheets, the underlying numerical data and statistical analysis for Figure panels 1B, 2C, 2D, 2E, 3A, 3B, 4C, 4E, 5A, 5B, 6A, 6B, 6C, 6D, 7C, 7F, S1A, S1C, S2B, S3, S4, S5B, S5E, S6D, S7A, S7B, S7C, S7D, S7E, S8B and S9B Figs. (XLSX)

S1 FCSfile FCS_FACS_ file for lipofectamine flow cytometry data in S8B Fig. FCS_FACS_, flow cytometry standard.
(FCS)

S2 FCSfile FCS_FACS_ file for LAG3 flow cytometry data in S8B Fig. FCS_FACS_, flow cytometry standard; LAG3, lymphocyte activation gene 3
(FCS)

S3 FCSfile FCS_FACS_ file for neurexin 1β flow cytometry data in S8B Fig. FCS_FACS_, flow cytometry standard.
(FCS)

S1 Fig Characterization of monomer and PFF αS. (A) MALDI-TOF mass spectrometry was used to confirm the presence of the N-terminal acetyl group as well as the purity of the samples for both unlabeled and AL488-labeled αS. The expected masses for αS_acetyl_^E130C^ and αS_acetyl_^E130C^-AL488 are 14,576 and 15,174, respectively; for αS_un_^E130C^ and αS_un_^E130C^-AL488, they are 14,434 and 15,132, respectively; the reported values are within expected accuracy for MALDI-TOF. (B) The fibril morphology before and after sonication was examined by TEM at 100,000x magnification. Scale bar = 200 μm.(C) Frequency distribution of PFF length following sonication; 50 fibers were measured, with an average length of 192.2 ± 56.9 nm for αS_acetyl_ and 209.7 ± 110.2 nm for αS_un_. (D) PAGE analysis at the end of the aggregation assay indicates that very little monomer αS is present in PFF preparations. 1 = molecular weight standards; 2 = αS_acetyl_ pellet; 3 = αS_acetyl_ supernatant; 4 = αS_un_ pellet; 5 = αS_un_ supernatant. The underlying data for this figure can be found in S1 Data.
(TIF)

S2 Fig Time-dependent endocytosis of αSacetyl monomer and PFFs. (A) Time-dependent uptake of αS_acetyl_ (green) monomer or PFFs by untreated or PNGase F–treated SH-SY5Y cells. Incubation time indicated above each image. Cells were stained with LysoTracker Deep Red (purple) prior to imaging. (B) Image overlap statistics for (A) of LysoTracker and αS_acetyl_ monomer and PFFs at the indicated incubation time. Colocalization was analyzed with the Pearson correlation coefficient. A larger coefficient reflects more overlap between αS_acetyl_-AL488 puncta and LysoTracker puncta (endosomes). Correlation coefficient was computed using the ImageJ plugin for colocalization (*n* = 100 cells, 3 independent experiments). Scale bars = 20 μm. The underlying data for this figure can be found in S1 Data.
(TIF)

S3 Fig Quantification of cell uptake measured by PAGE analysis. Uptake of αS_acetyl_ monomer (200 nM αS_acetyl_-AL488) or PFFs (200 nM in monomer units, 20:1 αS_acetyl_:αS_acetyl_-AL488) by SH-SY5Y cells as measured by PAGE analysis with fluorescence imaging of the gels (to detect only αS_acetyl_-AL488). (Upper) Gels show αS_acetyl_ remaining in the media at the time points indicated above the gels. Uptake is measured by quantifying the decrease of αS_acetyl_-AL488 in the media as a function of time. Quantification of the gels is shown as the scatter plot for monomer and PFF αS_acetyl_ +/− PNGase F treatment. The measurements are analogous to the FCS measurements shown in Fig 2C in the main manuscript, and the results of both approaches are comparable. (Lower) Gels show αS_acetyl_ internalized by cells at the time points indicated above the gels. Uptake is measured by quantifying the amount of αS_acetyl_-AL488 from lysed cells as a function of time. Transferrin-AL488, which exhibits very rapid uptake kinetics (S4C Fig), was added to cells for 30 minutes prior to lysis, to be used as loading control. The scatter plot compares the amount of internalized monomer and PFF αS_acetyl_, and the bar plot compares the amount of both forms internalized +/− PNGase F treatment. Quantification of gel band intensity was computed using ImageJ. These measurements are analogous to the image analysis shown in Fig 2C, and the results from both approaches are comparable. For each experiment, 3 independent measurements were made. The underlying data for this figure can be found in S1 Data.
(TIF)

S4 Fig Clathrin-dependent endocytosis. (A) Inhibition of endocytosis monitored by uptake of αS_acetyl_ monomer or PFFs at 4°C. Images are shown of uptake of protein at 37°C and 4°C for each condition. Protein was added to the cells followed by incubation at 4°C for 30 minutes. The controls were then moved to 37°C incubator. The two groups of cells were incubated in the respective temperatures for an additional 4 hours. (B) HEK cells incubated with αS _acetyl_ monomer or PFFs for 12 hours. (C) Rates of clathrin-dependent endocytosis of SH-SH5Y and HEK cells compared by loss of 100 nM transferrin-AL488 from extracellular medium of cells as measured by FCS. All αS_acetyl_ uptake measurements used 200 nM αS-AL488 monomer or PFF (concentration in monomer units, 20:1 αS:αS-AL488) Scale bar = 20μm. The underlying data for this figure can be found in S1 Data.
(TIF)

S5 Fig Treatment of cells with endoglycosidases. (A) Heparinase treatment of SH-SY5Y cells inhibits uptake of αS_un_ PFF (upper) but not of αS_acetyl_ PFF (lower). Images made after 12 hours of incubation of SH-SY5Y cells with 200 nM PFF αS_acetyl_-AL488 or αS_un_-AL488 (concentration in monomer units, 20:1 αS:αS-AL488) following treatment with Heparinase. (B) Heparinase and Endo H treatments of SH-SY5Y cells do not inhibit uptake of monomer αS_acetyl_. Images were made after 12 hours of incubation of SH-SY5Y cells with monomer αS_acetyl_-AL488 (200 nM) following treatment with Endo H or Heparinase. Quantification of monomer αS_acetyl_ uptake by SH-SY5Y cells treated with Endo H or Heparinase shown relative to PNGase F–treated or–untreated cells (Fig 2E). Numbers of puncta were computed using the ImageJ plugin for particle analysis (*n* = 100 cells, 3 independent experiments, significance analyzed by Student *t* test. (C) 50 nM conA-AL488 incubated with SH-SY5Y cells +/− PNGase F treatment. A significant reduction in the amount of conA-AL488 is observed in cells treated with PNGase F. (D) (Upper) Uptake of transferrin and αS_acetyl_ by PNGase F–treated SH-SY5Y cells. Internalization of transferrin-AL488 is not impacted by this treatment, indicating that clathrin-mediated endocytic pathways are functional. Uptake of αS_acetyl_-AL594 is significantly reduced. (Lower) As with the SH-SY5Y cells, uptake of transferrin by primary neurons from embryonic mouse hippocampus is not impacted by PNGase F treatment, indicating that clathrin-mediated endocytic pathways are functional. (E) Colorimetric measure of toxicity following the incubation of SH-SY5Y cells with 200 nM αS_acetyl_ monomer or PFFs (concentration in monomer units) for times indicated on plots. Data are expressed relative to vehicle-only addition. Each histogram bar is the average of 8 on-plate repeats across each of 3 independently performed replicates (*n* = 24). The underlying data for this figure can be found in S1 Data.
(TIF)

S6 Fig αS binding to and clustering of SH-SY5Y GPMVs is dependent on N-linked glycans. (A) Representative images of SH-SY5Y GPMVs incubated with 100 nM αS_acetyl_-AL488 and 80 μM of unlabeled αS_acetyl_. (B) Representative images of SH-SY5Y GPMVs incubated with 100 nM of αS_acetyl_-AL488 PFFs (monomer units, 20:1 αS:αS-AL488). (C) As in (B) but with with αS_un_-AL488 PFFs. (D) Intensity per pixel of 100 nM αS_acetyl_-AL488 and αS_un_-AL488 and 80 μM of unlabeled protein bound to GPMVs with and without PNGase F treatment. (E) Representative images of PNGase F–treated SH-SY5Y GPMVs incubated with 100 nM αS-AL488 and 80 μM of unlabeled αS. (F) Representative images of Endo H- and Heparinase-treated SH-SY5Y GPMVs incubated with 100 nM αS_acetyl_-AL488 and 80 μM of unlabeled αS_acetyl_. (G) Representative images of HEK GPMVs incubated with 100 nM αS_acetyl_-AL488 and varying concentrations of unlabeled αS_acetyl_ (indicated). (H) GPMVs incubated with 50 nM conA-AL488 or 50 nM wheat germ agglutinin-AL488, +/− PGNase F treatment (upper/lower). (I) GPMVs incubated with 100 nM αS_acetyl_-AL594 and 80 μM of unlabeled αS_acetyl_ prior to addition of 50 nM conA-AL488. (J) GPMVs incubated with 80 μM of unlabeled αS_acetyl_ prior to the addition of 50 nM conA-AL488. For all experiments, GPMVs equivalent to 5 μM total lipid as measured by the phosphate assay were used. Scale bars = 10 μm. The underlying data for this figure can be found in S1 Data.
(TIF)

S7 Fig αS binding to isolated glycans. (A) Averaged autocorrelation curves (30 curves of 10 seconds each) and fits to Eq 3 for αS_acetyl_ in the presence and absence of PNGase F–cleaved glycans. (B) The number of αS_acetyl_ molecules, *N*, upon, titration with PNGase F–cleaved glycans by FCS (same measurements as analyzed for diffusion time in Fig 6A). A decrease in *N* as a function of glycan concentration would reflect aggregation or oligomerization of the protein; this is not seen here. (C) Diffusion time of 80 nM conA-AL488 as a function of increasing concentrations of PNGase F–derived glycans. (D) Diffusion time of 80 nM αS_acetyl_-AL488 as a function of increasing concentrations of Endo H- and Heparinase-derived glycans. Data for PNGase F–derived glycans also shown for comparison (Fig 6A). (E) Quantification of PNGase F–cleaved glycans bound to monomer and PFF forms of αS_acetyl_ using total carbohydrate assay (reported as absorption at 490 nm). For comparison, positive-control conA is shown. All results are relative to the initial glycan pool, which is treated to the same filtration and quantification protocol as the samples. Details of the assay are described in the Materials and methods. The underlying data for this figure can be found in S1 Data.
(TIF)

S8 Fig Internalization of αS by HEK cells transfected with LAG3 or neurexin 1β. (A) HEK cells transfected with eGFP-tagged LAG3 (upper) or eGFP-tagged neurexin 1β (lower), imaged 48 hours after transfection. (B) Transfection efficiencies of eGFP-tagged neurexin 1β (upper: approximately 21%) or eGFP-tagged LAG3 (lower: approximately 16%) measured by flow cytometry. Lipofectamine-only sample was used as an eGFP negative control used for gating purposes in the analysis. (C) HEK cells transfected with eGFP-tagged neurexin 1β as in (A) but with the addition of αS_acetyl_-AL594 or αS_un_-AL594 PFFs. Cells were incubated with 200 nM PFFs (concentration in monomer units, 1:20 labeled:unlabeled) for 1 hour prior to imaging. To visualize the binding of PFFs to the extracellular membrane, no Trypan blue solution was used in these experiments. Scale bars = 20 μm. The underlying data for this figure can be found in S1 Data and S1 FCSfile, S2 FCSfile, and S3 FCSfile.
(TIF)

S9 Fig PNGase F does not remain bound to cell membranes after incubation. (A) The amount of PNGase F before and after treatment of GPMVs and SH-SY5Y cells was examined by PAGE. The majority of the enzyme is recovered from the GPMV buffer or cell media, indicating that it does not remain bound to the membranes, potentially blocking αS_acetyl_ binding. (B) αS_acetyl_ is stable during incubation with cell media during uptake studies. FCS was used to monitor the diffusion time of αS_acetyl_ in media as a function of time during incubation with SH-SY5Y cells. The diffusion time is stable over the 24-hour period, indicating that the protein is not degraded nor is the fluorophore cleaved, both of which would be expected to result in a faster diffusion time. (C) SH-SY5Y GPMVs and cells do not bind or uptake eGFP, respectively. eGFP was used as negative control. The addition of 80 nM eGFP to GPMVs shows no evidence of binding. Likewise, there is no evidence of uptake of eGFP by SH-SY5Y cells following 12 hours of incubation. Scale bar = 20 μm. The underlying data for this figure can be found in S1 Data.
(TIF)

## Abbreviations

αS: α-Synuclein
αS-pS129: αS phosphorylated at serine 129
αSacetyl: N-terminally acetylated αS
αSun: unmodified αS
AL488: Alexa Fluor 488
AL594: Alexa Fluor 594
αS-AL488: αS fluorescently labeled with AL488
αS-AL594: αS fluorescently labeled with AL594
con A: concanavalin A
conA-AL488: AL488-labeled conA
DMEM: Dulbecco's Modified Eagle's Medium
eGFP: enhanced green fluorescent protein
Endo H: endoglycosidase H
ET_eff_: energy transfer efficiency
FCS_FACS_: flow cytometry standard
FCS: fluorescence correlation spectroscopy
FLIM: fluorescence lifetime imaging microscopy
FRET: Förster resonance energy transfer
GdmCl: guanidine hydrochloride
GlcNAc: N-acetylglycosamine
GPMV: giant plasma membrane vesicle
H-Tri: H-Trisaccharide
HEK: human embryonic kidney 293T
HSQC: heteronuclear single quantum coherence
IACUC: Institutional Animal Care and Use Committee
LAG3: lymphocyte activation gene 3
MALDI: matrix-assisted laser desorption/ionization
NatB: N-terminal acetyltransferase B
NMR: nuclear magnetic resonance
PFF: preformed fibril
PNGase F: peptide-N-glycosidase F
PTNC: Penn Medicine Translational Neuroscience Center
SH-SY5Y: human neuroblastoma
SPAD: sensitive avalanche photodiode detector
