# Supplementary figures and images for: Identification of N-linked glycans as specific mediators of neuronal uptake of acetylated α-Synuclein
Source: PLoS Biol. 2019 Jun 18;17(6):e3000318. doi: 10.1371/journal.pbio.3000318 (PMC6599126; doi:10.1371/journal.pbio.3000318)

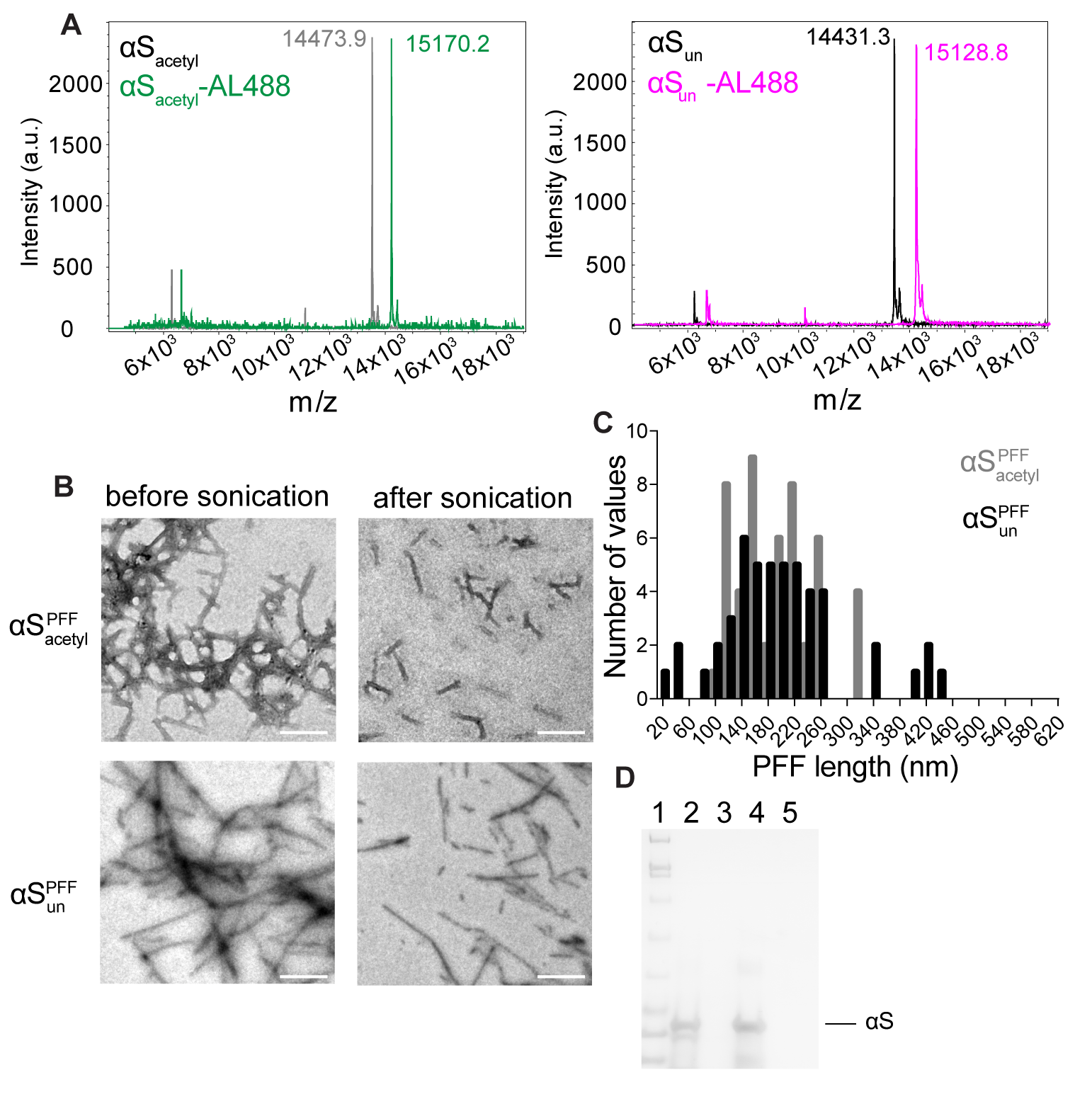

Supplement: S1 Fig — (A) MALDI-TOF mass spectrometry was used to confirm the presence of the N-terminal acetyl group as well as the purity of the samples for both unlabeled and AL488-labeled αS. The expected masses for αSacetylE130C and αSacetylE130C-AL488 are 14,576 and 15,174, respectively; for αSunE130C and αSunE130C-AL488, they are 14,434 and 15,132, respectively; the reported values are within expected accuracy for MALDI-TOF. (B) The fibril morphology before and after sonication was examined by TEM at 100,000x magnification. Scale bar = 200 μm.(C) Frequency distribution of PFF length following sonication; 50 fibers were measured, with an average length of 192.2 ± 56.9 nm for αSacetyl and 209.7 ± 110.2 nm for αSun. (D) PAGE analysis at the end of the aggregation assay indicates that very little monomer αS is present in PFF preparations. 1 = molecular weight standards; 2 = αSacetyl pellet; 3 = αSacetyl supernatant; 4 = αSun pellet; 5 = αSun supernatant. The underlying data for this figure can be found in S1 Data. (TIF) [file pbio.3000318.s005.tif]

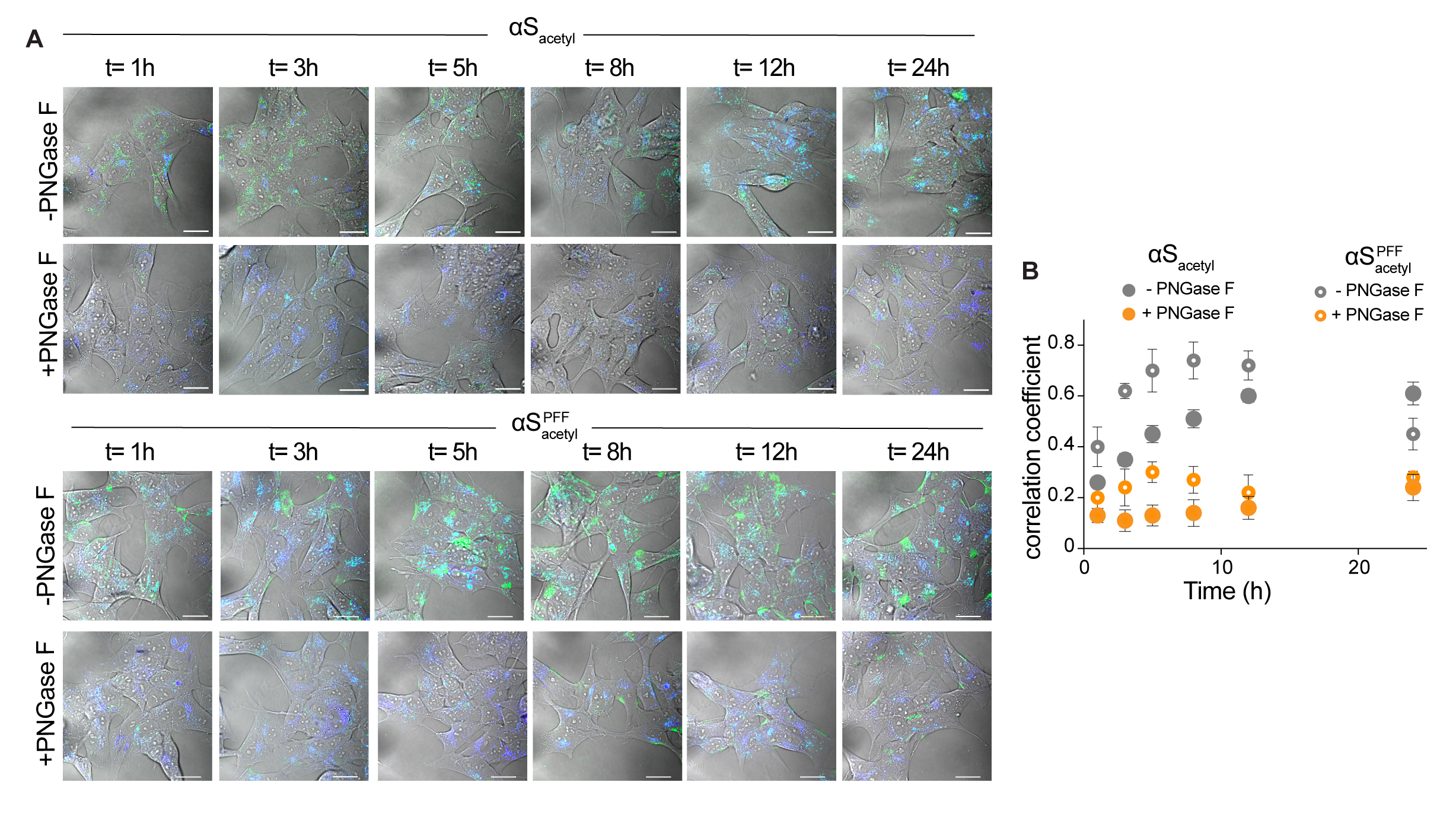

Supplement: S2 Fig — (A) Time-dependent uptake of αSacetyl (green) monomer or PFFs by untreated or PNGase F–treated SH-SY5Y cells. Incubation time indicated above each image. Cells were stained with LysoTracker Deep Red (purple) prior to imaging. (B) Image overlap statistics for (A) of LysoTracker and αSacetyl monomer and PFFs at the indicated incubation time. Colocalization was analyzed with the Pearson correlation coefficient. A larger coefficient reflects more overlap between αSacetyl-AL488 puncta and LysoTracker puncta (endosomes). Correlation coefficient was computed using the ImageJ plugin for colocalization (n = 100 cells, 3 independent experiments). Scale bars = 20 μm. The underlying data for this figure can be found in S1 Data. (TIF) [file pbio.3000318.s006.tif]

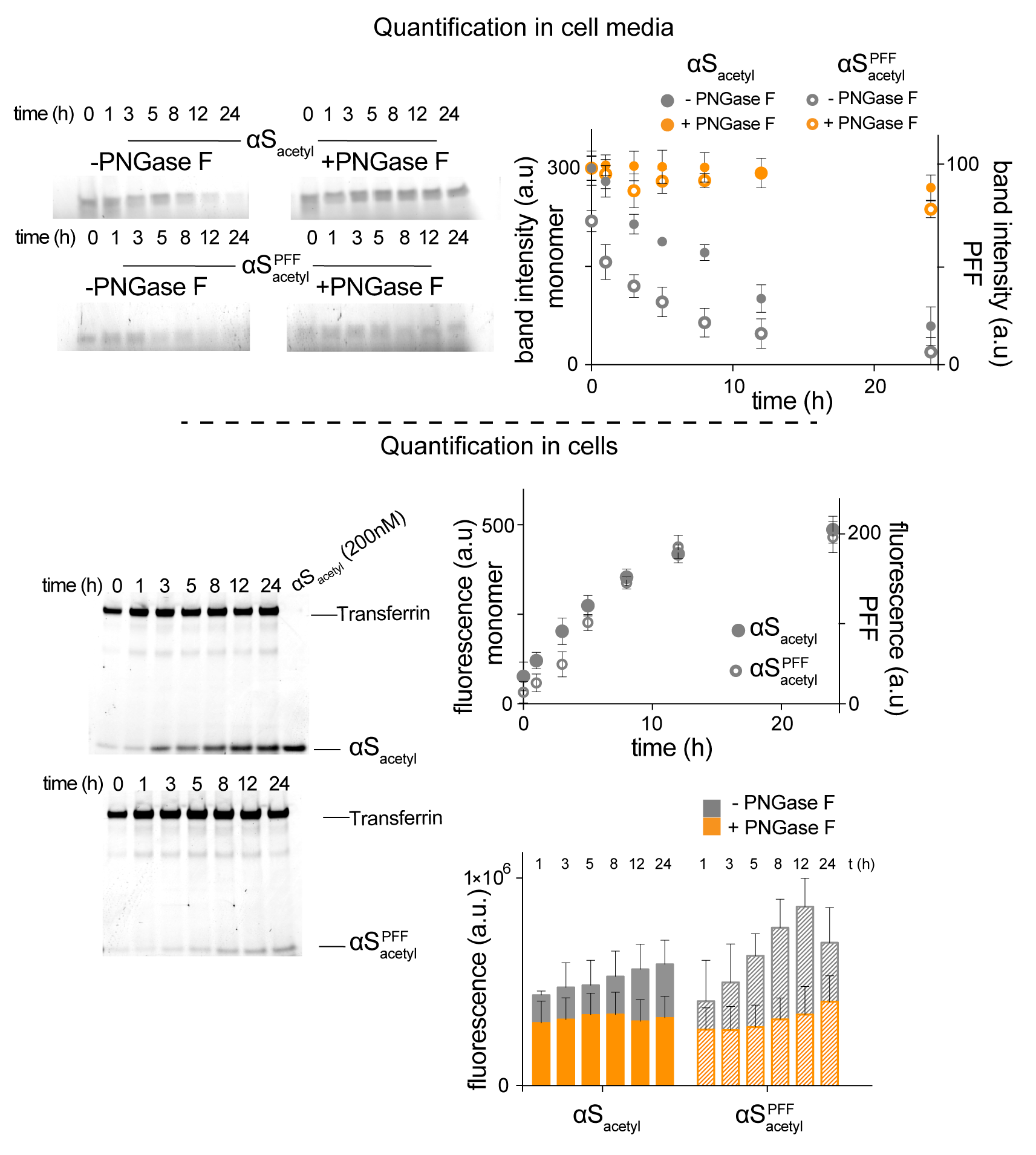

Supplement: S3 Fig — Uptake of αSacetyl monomer (200 nM αSacetyl-AL488) or PFFs (200 nM in monomer units, 20:1 αSacetyl:αSacetyl-AL488) by SH-SY5Y cells as measured by PAGE analysis with fluorescence imaging of the gels (to detect only αSacetyl-AL488). (Upper) Gels show αSacetyl remaining in the media at the time points indicated above the gels. Uptake is measured by quantifying the decrease of αSacetyl-AL488 in the media as a function of time. Quantification of the gels is shown as the scatter plot for monomer and PFF αSacetyl +/− PNGase F treatment. The measurements are analogous to the FCS measurements shown in Fig 2C in the main manuscript, and the results of both approaches are comparable. (Lower) Gels show αSacetyl internalized by cells at the time points indicated above the gels. Uptake is measured by quantifying the amount of αSacetyl-AL488 from lysed cells as a function of time. Transferrin-AL488, which exhibits very rapid uptake kinetics (S4C Fig), was added to cells for 30 minutes prior to lysis, to be used as loading control. The scatter plot compares the amount of internalized monomer and PFF αSacetyl, and the bar plot compares the amount of both forms internalized +/− PNGase F treatment. Quantification of gel band intensity was computed using ImageJ. These measurements are analogous to the image analysis shown in Fig 2C, and the results from both approaches are comparable. For each experiment, 3 independent measurements were made. The underlying data for this figure can be found in S1 Data. (TIF) [file pbio.3000318.s007.tif]

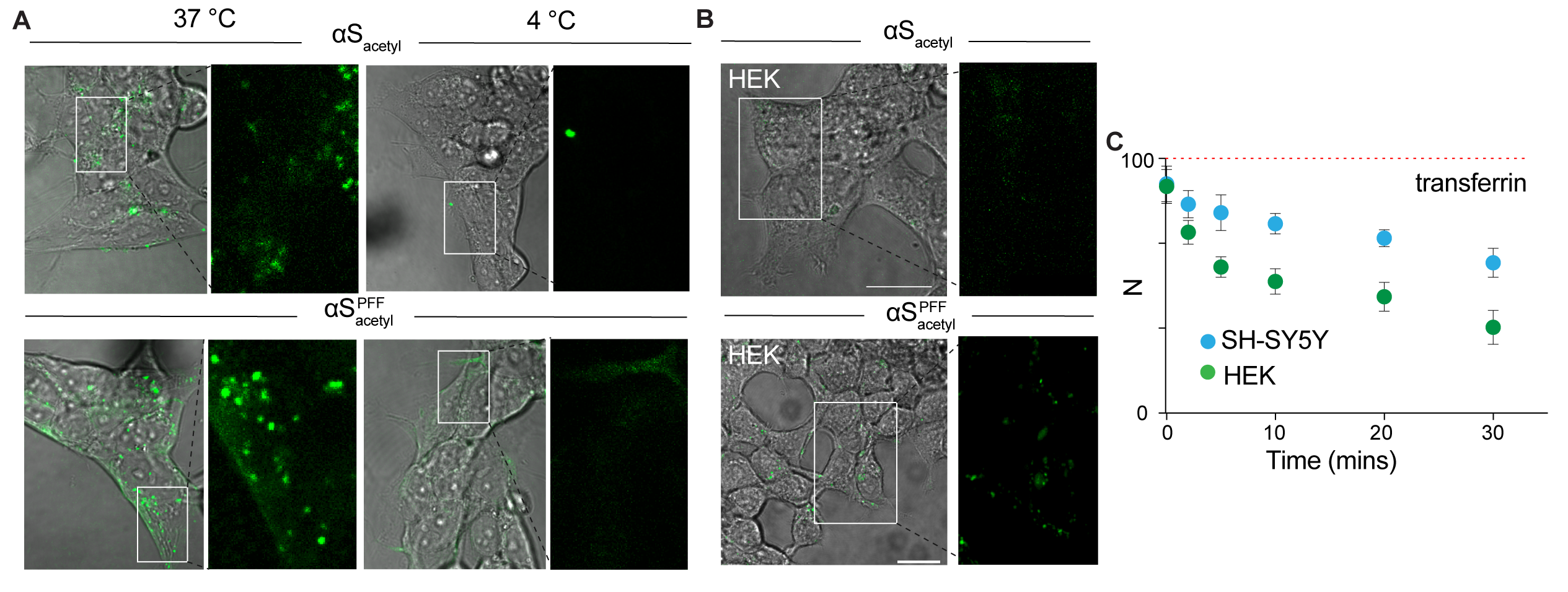

Supplement: S4 Fig — (A) Inhibition of endocytosis monitored by uptake of αSacetyl monomer or PFFs at 4°C. Images are shown of uptake of protein at 37°C and 4°C for each condition. Protein was added to the cells followed by incubation at 4°C for 30 minutes. The controls were then moved to 37°C incubator. The two groups of cells were incubated in the respective temperatures for an additional 4 hours. (B) HEK cells incubated with αS acetyl monomer or PFFs for 12 hours. (C) Rates of clathrin-dependent endocytosis of SH-SH5Y and HEK cells compared by loss of 100 nM transferrin-AL488 from extracellular medium of cells as measured by FCS. All αSacetyl uptake measurements used 200 nM αS-AL488 monomer or PFF (concentration in monomer units, 20:1 αS:αS-AL488) Scale bar = 20μm. The underlying data for this figure can be found in S1 Data. (TIF) [file pbio.3000318.s008.tif]

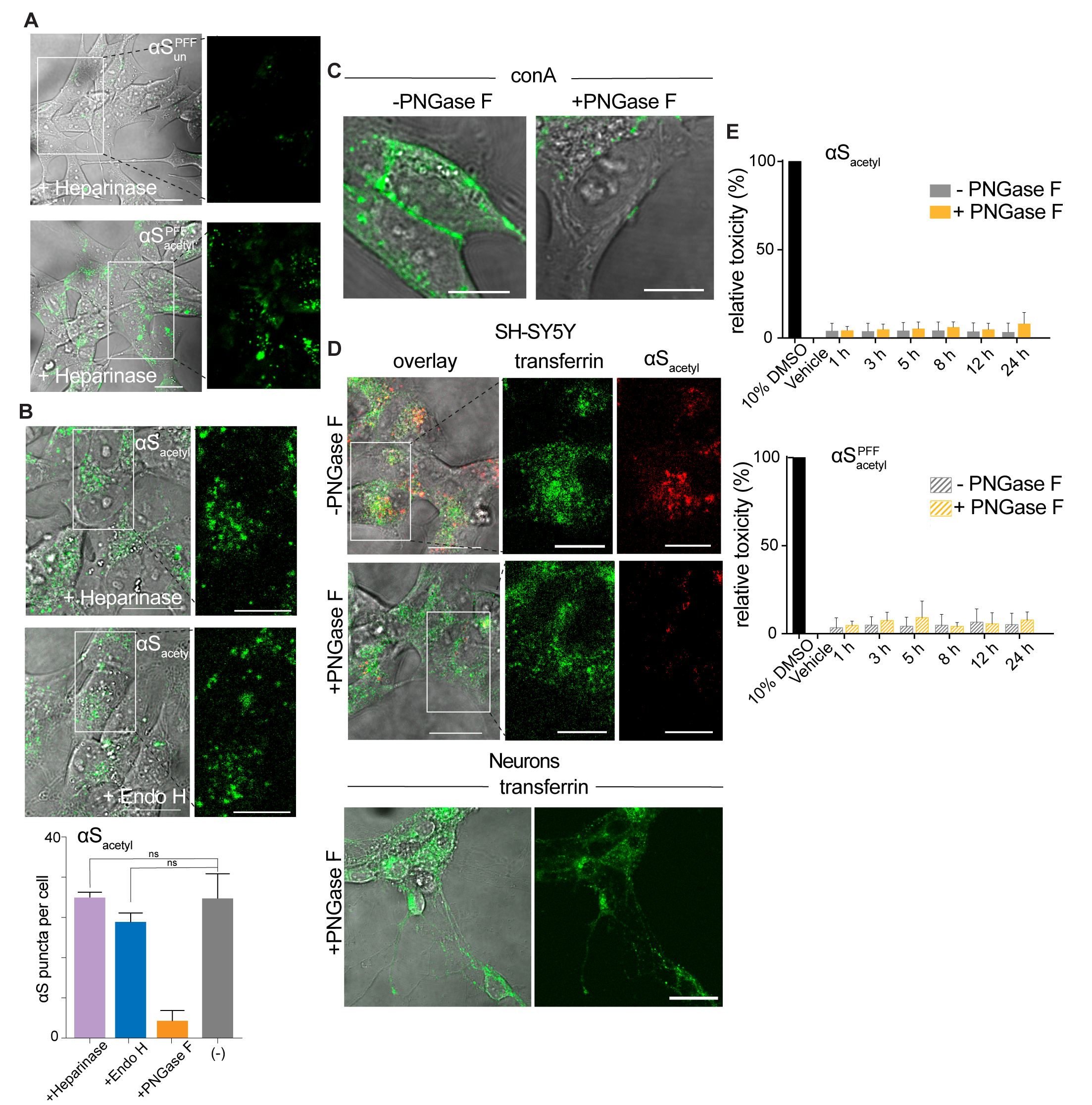

Supplement: S5 Fig — (A) Heparinase treatment of SH-SY5Y cells inhibits uptake of αSun PFF (upper) but not of αSacetyl PFF (lower). Images made after 12 hours of incubation of SH-SY5Y cells with 200 nM PFF αSacetyl-AL488 or αSun-AL488 (concentration in monomer units, 20:1 αS:αS-AL488) following treatment with Heparinase. (B) Heparinase and Endo H treatments of SH-SY5Y cells do not inhibit uptake of monomer αSacetyl. Images were made after 12 hours of incubation of SH-SY5Y cells with monomer αSacetyl-AL488 (200 nM) following treatment with Endo H or Heparinase. Quantification of monomer αSacetyl uptake by SH-SY5Y cells treated with Endo H or Heparinase shown relative to PNGase F–treated or–untreated cells (Fig 2E). Numbers of puncta were computed using the ImageJ plugin for particle analysis (n = 100 cells, 3 independent experiments, significance analyzed by Student t test. (C) 50 nM conA-AL488 incubated with SH-SY5Y cells +/− PNGase F treatment. A significant reduction in the amount of conA-AL488 is observed in cells treated with PNGase F. (D) (Upper) Uptake of transferrin and αSacetyl by PNGase F–treated SH-SY5Y cells. Internalization of transferrin-AL488 is not impacted by this treatment, indicating that clathrin-mediated endocytic pathways are functional. Uptake of αSacetyl-AL594 is significantly reduced. (Lower) As with the SH-SY5Y cells, uptake of transferrin by primary neurons from embryonic mouse hippocampus is not impacted by PNGase F treatment, indicating that clathrin-mediated endocytic pathways are functional. (E) Colorimetric measure of toxicity following the incubation of SH-SY5Y cells with 200 nM αSacetyl monomer or PFFs (concentration in monomer units) for times indicated on plots. Data are expressed relative to vehicle-only addition. Each histogram bar is the average of 8 on-plate repeats across each of 3 independently performed replicates (n = 24). The underlying data for this figure can be found in S1 Data. (TIF) [file pbio.3000318.s009.tif]

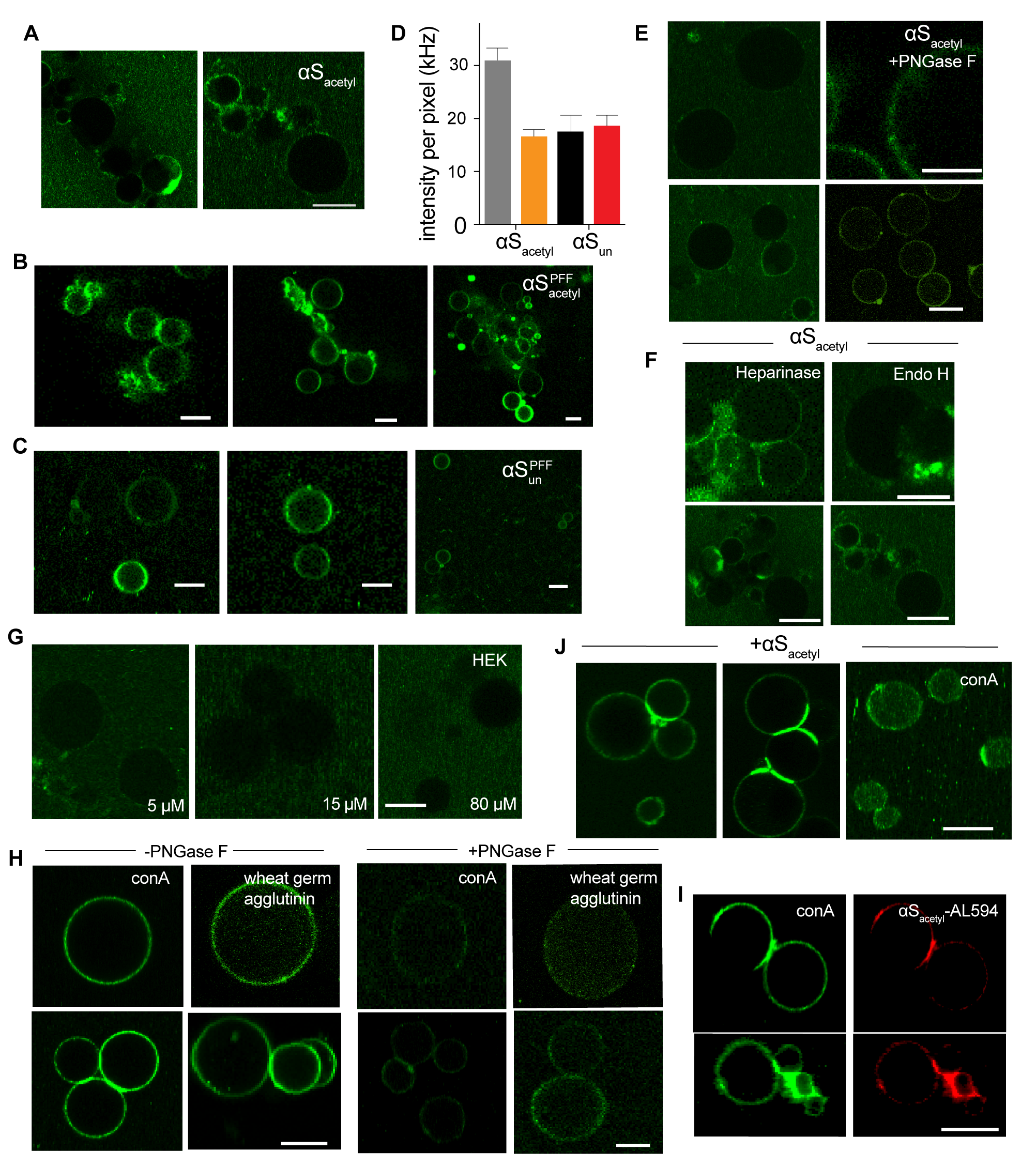

Supplement: S6 Fig — (A) Representative images of SH-SY5Y GPMVs incubated with 100 nM αSacetyl-AL488 and 80 μM of unlabeled αSacetyl. (B) Representative images of SH-SY5Y GPMVs incubated with 100 nM of αSacetyl-AL488 PFFs (monomer units, 20:1 αS:αS-AL488). (C) As in (B) but with with αSun-AL488 PFFs. (D) Intensity per pixel of 100 nM αSacetyl-AL488 and αSun-AL488 and 80 μM of unlabeled protein bound to GPMVs with and without PNGase F treatment. (E) Representative images of PNGase F–treated SH-SY5Y GPMVs incubated with 100 nM αS-AL488 and 80 μM of unlabeled αS. (F) Representative images of Endo H- and Heparinase-treated SH-SY5Y GPMVs incubated with 100 nM αSacetyl-AL488 and 80 μM of unlabeled αSacetyl. (G) Representative images of HEK GPMVs incubated with 100 nM αSacetyl-AL488 and varying concentrations of unlabeled αSacetyl (indicated). (H) GPMVs incubated with 50 nM conA-AL488 or 50 nM wheat germ agglutinin-AL488, +/− PGNase F treatment (upper/lower). (I) GPMVs incubated with 100 nM αSacetyl-AL594 and 80 μM of unlabeled αSacetyl prior to addition of 50 nM conA-AL488. (J) GPMVs incubated with 80 μM of unlabeled αSacetyl prior to the addition of 50 nM conA-AL488. For all experiments, GPMVs equivalent to 5 μM total lipid as measured by the phosphate assay were used. Scale bars = 10 μm. The underlying data for this figure can be found in S1 Data. (TIF) [file pbio.3000318.s010.tif]

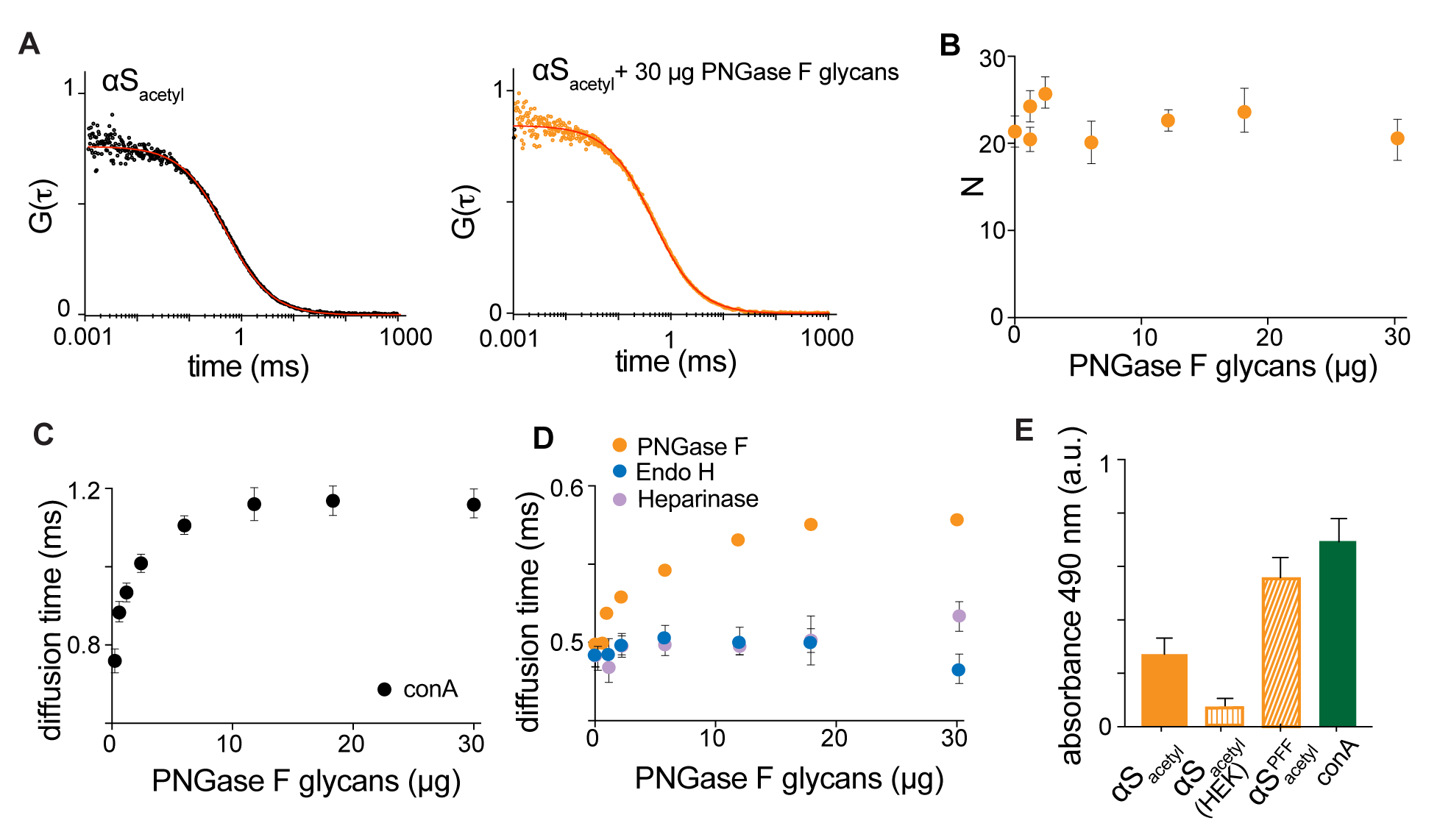

Supplement: S7 Fig — (A) Averaged autocorrelation curves (30 curves of 10 seconds each) and fits to Eq 3 for αSacetyl in the presence and absence of PNGase F–cleaved glycans. (B) The number of αSacetyl molecules, N, upon, titration with PNGase F–cleaved glycans by FCS (same measurements as analyzed for diffusion time in Fig 6A). A decrease in N as a function of glycan concentration would reflect aggregation or oligomerization of the protein; this is not seen here. (C) Diffusion time of 80 nM conA-AL488 as a function of increasing concentrations of PNGase F–derived glycans. (D) Diffusion time of 80 nM αSacetyl-AL488 as a function of increasing concentrations of Endo H- and Heparinase-derived glycans. Data for PNGase F–derived glycans also shown for comparison (Fig 6A). (E) Quantification of PNGase F–cleaved glycans bound to monomer and PFF forms of αSacetyl using total carbohydrate assay (reported as absorption at 490 nm). For comparison, positive-control conA is shown. All results are relative to the initial glycan pool, which is treated to the same filtration and quantification protocol as the samples. Details of the assay are described in the Materials and methods. The underlying data for this figure can be found in S1 Data. (TIF) [file pbio.3000318.s011.tif]

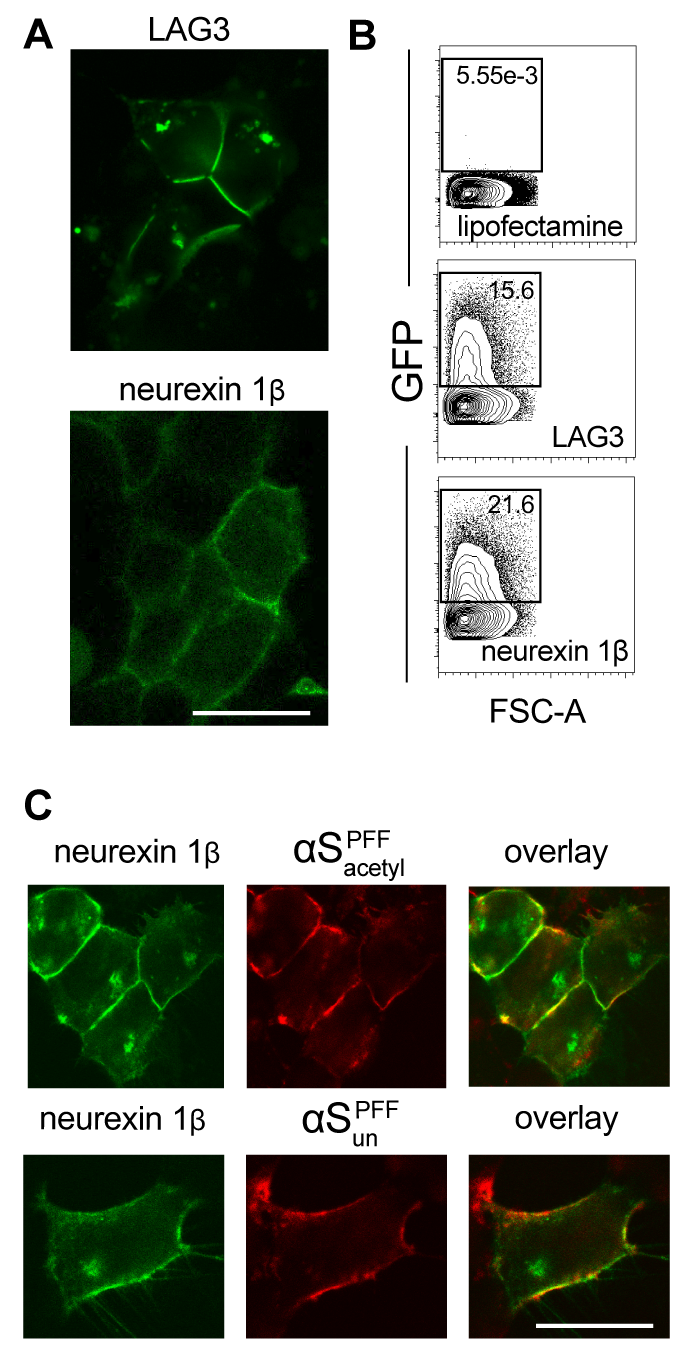

Supplement: S8 Fig — (A) HEK cells transfected with eGFP-tagged LAG3 (upper) or eGFP-tagged neurexin 1β (lower), imaged 48 hours after transfection. (B) Transfection efficiencies of eGFP-tagged neurexin 1β (upper: approximately 21%) or eGFP-tagged LAG3 (lower: approximately 16%) measured by flow cytometry. Lipofectamine-only sample was used as an eGFP negative control used for gating purposes in the analysis. (C) HEK cells transfected with eGFP-tagged neurexin 1β as in (A) but with the addition of αSacetyl-AL594 or αSun-AL594 PFFs. Cells were incubated with 200 nM PFFs (concentration in monomer units, 1:20 labeled:unlabeled) for 1 hour prior to imaging. To visualize the binding of PFFs to the extracellular membrane, no Trypan blue solution was used in these experiments. Scale bars = 20 μm. The underlying data for this figure can be found in S1 Data and S1 FCSfile, S2 FCSfile, and S3 FCSfile. (TIF) [file pbio.3000318.s012.tif]

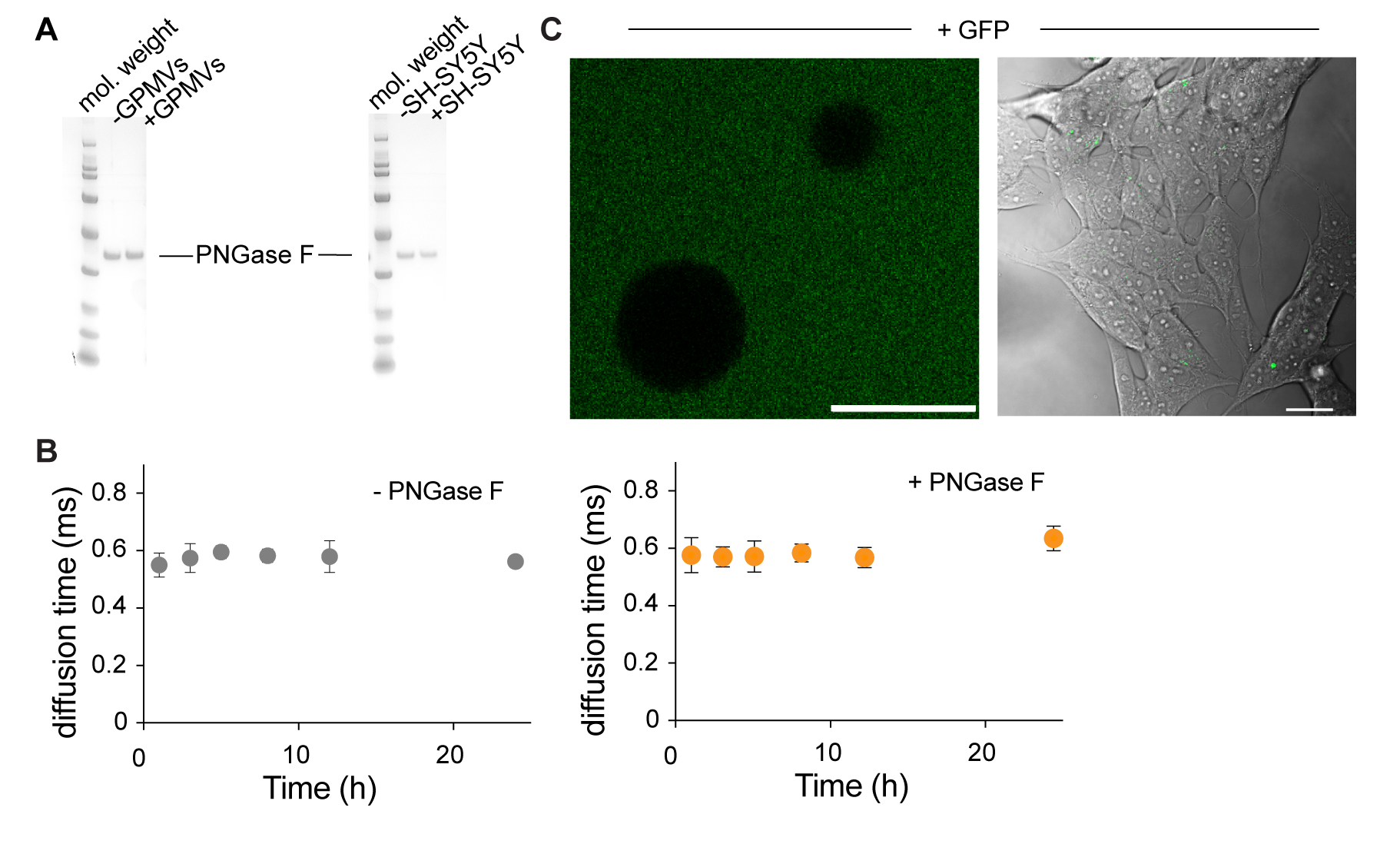

Supplement: S9 Fig — (A) The amount of PNGase F before and after treatment of GPMVs and SH-SY5Y cells was examined by PAGE. The majority of the enzyme is recovered from the GPMV buffer or cell media, indicating that it does not remain bound to the membranes, potentially blocking αSacetyl binding. (B) αSacetyl is stable during incubation with cell media during uptake studies. FCS was used to monitor the diffusion time of αSacetyl in media as a function of time during incubation with SH-SY5Y cells. The diffusion time is stable over the 24-hour period, indicating that the protein is not degraded nor is the fluorophore cleaved, both of which would be expected to result in a faster diffusion time. (C) SH-SY5Y GPMVs and cells do not bind or uptake eGFP, respectively. eGFP was used as negative control. The addition of 80 nM eGFP to GPMVs shows no evidence of binding. Likewise, there is no evidence of uptake of eGFP by SH-SY5Y cells following 12 hours of incubation. Scale bar = 20 μm. The underlying data for this figure can be found in S1 Data. (TIF) [file pbio.3000318.s013.tif]
